# How to make causal inferences using texts

**DOI:** 10.1126/sciadv.abg2652

**Published:** 2022-10-19

**Authors:** Naoki Egami, Christian J. Fong, Justin Grimmer, Margaret E. Roberts, Brandon M. Stewart

**Affiliations:** ^1^ Department of Political Science, Columbia University, New York, NY 10027, USA.; ^2^ Department of Political Science, University of Michigan, Ann Arbor, MI 48109, USA.; ^3^ Department of Political Science, Stanford University, Stanford, CA 94305, USA.; ^4^ Hoover Institution, Stanford University, Stanford, CA 94305, USA.; ^5^ Department of Political Science, University of California San Diego, La Jolla, CA 92093, USA.; ^6^ Halıcıoğlu Data Science Institute, University of California San Diego, La Jolla, CA 92093, USA.; ^7^ Department of Sociology, Princeton University, Princeton, NJ 08544, USA.; ^8^ Office of Population Research, Princeton University, Princeton, NJ 08544, USA.

## Abstract

Text as data techniques offer a great promise: the ability to inductively discover measures that are useful for testing social science theories with large collections of text. Nearly all text-based causal inferences depend on a latent representation of the text, but we show that estimating this latent representation from the data creates underacknowledged risks: we may introduce an identification problem or overfit. To address these risks, we introduce a split-sample workflow for making rigorous causal inferences with discovered measures as treatments or outcomes. We then apply it to estimate causal effects from an experiment on immigration attitudes and a study on bureaucratic responsiveness.

## Teaser

Identification challenges in causal inference with documents can be addressed with sample splitting.

## INTRODUCTION

Social scientists increasingly use text-based measures as dependent or independent variables (*1*–*6*). Texts are complex, high-dimensional objects; thus, researchers must find simpler, lower-dimensional representations for their texts to use them in scientific analyses. This simplification can be intuitive and familiar. For example, we might take a collection of emails and divide them into “spam” and “not spam.” We call the function that maps the documents into our measure of interest *g*. *g* acts as a codebook that tells us how to compress our documents into categories, topics, or dimensions of interest.

Researchers rarely know *g* before they have seen their data. Instead, they discover it inductively from the data itself. This includes hand-coding and supervised methods that start with predetermined categories and discover a mapping from features of the texts to those categories (*7*), clustering and topic models that discover an organization of texts and then assign documents to those categories (*8*), and factor analysis and item-response theory models that embed texts into a low-dimensional space (*9*). The need to discover and iteratively define measures and concepts from data is a fundamental component of social science research.

However, the iterative discovery process poses problems for causal inference. Standard causal inference frameworks, such as potential outcomes (*10*) and directed acyclic graphs (*11*), assume that the treatment and outcome are known and do not depend on the data. This produces well-defined causal estimands (*12*), but it contrasts with text-based causal inferences, where researchers’ *g* and, hence, the outcomes or treatments are often latent variables found from the data. Thus, when causal inference methods, including randomized experiments, are applied directly to text data, we suffer from distinct methodological challenges.

We connect the text as data literature (*13*–*15*), with the growing literature on causal inference in the social sciences (*11*, *16*) using a rigorous machine learning workflow for text-based causal inferences that focuses on the central role of discovery. Our workflow highlights an identification and estimation problem that arises from a common source—using the same documents to both discover measures and estimate causal effects. By using the same documents to discover *g* and estimate effects, the analyst creates a problem where the categories obtained depend on the particular randomization. Consequently, each randomization could create a different codebook function *g*. Because this different codebook function could contain categories with a different substantive interpretation or even a different number of categories, it is impossible to compare estimates across different randomizations. As a result, properties of estimators, such as bias, variance, and consistency, are not defined without further assumptions.

This problem is pervasive in the social sciences. Any time scholars work with some latent representation of data—either as treatments or outcomes—and then use those latent representations to make a causal inference, this problem is present unless scholars make additional assumptions or use a research design to eliminate its influence. Because of its pervasiveness, we call this problem the Fundamental Problem of Causal Inference with Latent Variables (FPCILV).

Even if we dismiss, or assume away, the identification problem, the complexity of text leads to an estimation problem when the same data are used to both estimate the codebook function and infer causal effects: overfitting. By using the same documents to discover and estimate effects, even well-intentioned analysts may mistake noise for a robust causal effect. The dangers of searching over *g* is a more general version of the problem of researchers recoding variables in an experiment to search for significance. This idea of overfitting also formalizes the intuition that some analysts have that latent-variable models are “baking in” an effect. Without using sample splitting, text as data causal inference research designs are exposed to a particularly dangerous kind of “fishing.” Not only can researchers search over specifications to find statistically significant results, without sample splitting they would also be able to alter the definition of their independent and dependent variables in search of whatever findings they wanted.

**Fig. 1.**
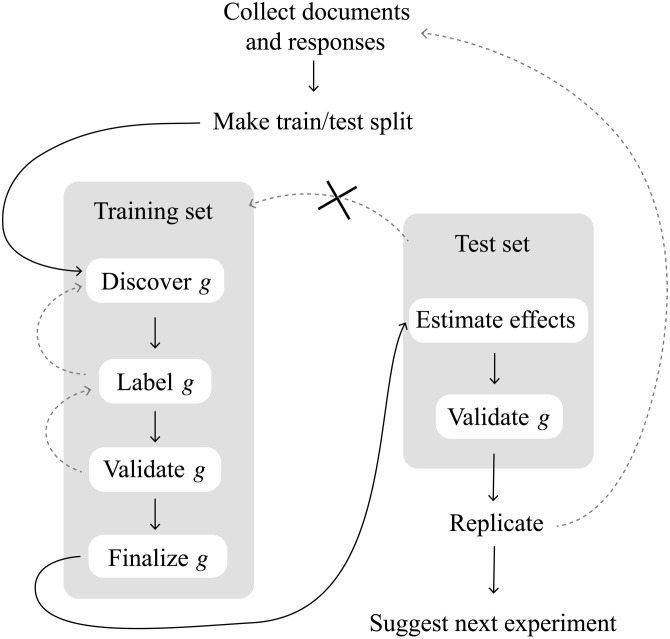
Our procedure for text-based causal inferences with latent treatments or outcomes.

Identification and overfitting problems caused by FPCILV can be addressed by using one dataset to discover the measures of interest and another to estimate causal effects. That way, a different randomization of the test set would not change *g*. Fortunately, it is not necessary to actually collect two separate datasets. Instead, researchers can simply divide one dataset into a training set for discovering measures and a test set for estimating causal effects (*17*). The estimate in the test set provides insight into what the results from a next experiment would be and, as we show below, resolves our identification and estimation problems. Figure 1 summarizes the procedure we recommend, and Supplement S4 provides a more detailed verbal description.

Of course, sample-splitting procedures are a fundamental and regularly used component of machine learning research, particularly for evaluating the performance of classifiers. Following a long tradition in statistics (*18*) and more recently in econometrics, it has been used to improve causal inference (*19*–*21*). However, this tradition has not engaged with the FPICLV or the overfitting problems that emerge when inferring *g* from data and then applying it to learn causal effects, so the value of sample-splitting to resolve these issues has not yet been appreciated.

To introduce this procedure, our paper proceeds as follows. We provide a definition of *g* and describe the central role it plays in text analysis. We then discuss the core identification and estimation concerns that complicate the use of *g* in a causal inference setting. We explain why sample splitting solves this problem, how it works, and the trade-offs in its use. We also defer discussion of prior work under this section so that we can show how our work connects to a long tradition of sample-splitting approaches in machine learning and, more recently, in causal inference. Last, we illustrate our approach using applications in two settings: text as outcome and text as treatment.

## RESULTS

The central problems that we address stem from the need to compress text data to facilitate causal inference. The codebook function, *g*, compresses high-dimensional text to a low-dimensional measure used for the treatment or outcome. In this section, we explain why *g* is essential, how to obtain *g*, and how to evaluate candidate *g*’s.

### What is *g* and why do we need it?

Documents are high-dimensional, complicated, and sparse; hence, text is typically not usable for social science inference in its raw form. Fortunately, social scientists are often interested in some emergent property of the text—such as the topic that is discussed, the sentiment expressed, or the ideological position taken. The result is that distinct blocks of text can convey similar topics or sentiment. Reducing the dimensions of the text allows us to group texts and make inferences from our data.

Suppose we are interested in understanding how candidate biographies influence the popularity of a candidate (*17*). Each biography is unique; thus, we cannot estimate the effect of any individual biography on a candidate’s popularity. Instead, we are interested in some latent property of the text’s effect on the popularity of the candidate, such as occupational background. In this example, *g* might compress the text of the biography into an indicator of whether the candidate is a lawyer. The analyst might define *g* by hand-coding, automatically from the text, by looking for the presence or absence of the word “lawyer,” or by a group of words or phrases that convey that someone has a legal background, such as “JD,” “attorney,” and “law school.” Being a lawyer is just one latent feature in the text. Different *g*’s might measure whether a candidate held prior office, went to college, or served in the military. These examples all map the text into binary categories, but *g* could also map into discrete categories, proportions, or continuous variables (like ideal point estimates).

Social scientists working on text as data have adopted this compression approach, although the low-dimensional representation is often only implicit (*8*, *9*, *13*). We can also think of *g* as the codebook function because it plays the role of a codebook in a manual content analysis, describing a procedure for organizing the researcher’s texts in some systematic way. *g* is always implicitly present whenever a set of documents is placed into a common set of categories or is assigned a common set of properties. *g* takes on a central role because it connects the raw text to the underlying property that the researcher cares about. Once a researcher decides on and estimates *g*, then text is usually ready to be used in statistical analysis.

#### Discovering g

While *g* is necessary to make causal inference, it is rarely known exactly from a theory or prior research. Instead, *g* is typically developed through iteration between coding rules and the documents to be coded. Even in manual content analysis (*22*), researchers typically read at least a portion of the documents to write a codebook that determines how coders should put documents into the categories of interest. The process is even more explicitly data-driven in automated content analysis.

There are three strategies for learning *g* from the data. First, we could read a sample of text. In manual content analysis, *g* often relies on some familiarity with the text or reading a sample of documents to decide how the text should map into categories. Second, we could use supervised learning, which is conceptually similar to manual content analysis, to infer *g* from hand-coded or otherwise labeled documents. Last, we could use unsupervised learning techniques to discover a low-dimensional representation.

#### Portability of g

When we fit a *g* within a sample, it is a mapping from words and features to labels. This might be extremely data specific, depending on the particular syntax and content with a particular corpus. This mapping, however, is intended to capture a more general concept or organization of the world. This organization, by construction, will exist outside of any one dataset and can be used in many distinct settings.

The concepts that we discover are portable, although the codebook function *g* will be corpus specific. When we are working inductively, we tend to use *g* to generate ideas about the conceptualization that is useful for making inferences. Once we have this concept, though, it is portable across different settings. The question in these other settings is how to construct a *g* to map from the features for that particular setting to the concept. In other words, we need a corpus-specific mapping, *g*, that corresponds with the general concept.

For example, consider a corpus of newspaper editorials. A researcher might be interested in a codebook, *g*, that maps the texts of the editorials to the concept of right-wing populism. If the editorials came from the contemporary United States, the codebook would probably look for features that expressed skepticism about immigration, affinity for protectionism, and affection for Donald Trump. To capture the same concept in the contemporary United Kingdom, researchers should instead use a codebook that focuses more on a distaste for the European Union. When the concept is captured with different codebooks, researchers must be cautious about making naïve comparisons of *g* across corpora. For example, a 0.9 in the United States means something different from a 0.9 in the United Kingdom. Therefore, when considering portability and external validity, researchers should focus on causal effects in terms of the underlying concept rather than a specific function *g* itself. If a researcher found that exposure to a populist editorial increases support for defense spending in the United States, they could test whether the relationship held in the United Kingdom as well. Here, researchers can conduct sign generalization (whether the sign of causal effects of exposure to right-wing populism is the same in the two countries) rather than effect generalization (whether point estimates are the same), which is a less relevant question because required codebook functions *g* are different.

### The problem of causal inference with *g*

The codebook function, *g*, encodes the mapping between the observed text and the low-dimensional representations we use to make inferences. In this section, we explain how this compression of information both facilitates and challenges causal inference with text. We first place *g* in the traditional causal inference setting. We then explain how the use of *g* leads to the FPCILV and overfitting.

#### Causal inference with g

To begin, we review potential outcomes notation and assumptions used when there is no text or dimensionality reduction, and we analyze a unidimensional treatment and outcome (*16*). Denote our dependent variable for each unit *i* [*i* ∈ (1,2, …, *N*)] with *Y_i_*; the treatment condition for unit *i* will be *T_i_*. We define the space of all possible outcomes as 𝒴 and the space of all possible treatment assignments as T. When the treatment is binary, we refer to *Y_i_*(1) as the potential outcome for unit *i* under treatment and *Y_i_*(0) as the potential outcome under control. The individual causal effect (ICE) for unit *i* is given by ICE*_i_* = *Y_i_*(1) − *Y_i_*(0). Our typical estimand is some function of the ICEs, such as the average treatment effect (ATE), *E*[*Y_i_*(1) − *Y_i_*(0)].

To identify the ATE using a randomized experiment, we make three key assumptions. First, we assume that the response depends only on the assigned treatment, often called the stable unit treatment value assumption (SUTVA). Specifically,

**Assumption 1** [SUTVA (*23*)]. *For each individual i, we assume that their response depends only on their assigned treatment status*.

Second, we assume that our treatment is randomly assigned:

**Assumption 2** (Ignorability). *Y_i_*(*t*) ⫫ *T_i_ for all t* ∈ T*. For each individual, we assume that their potential outcomes are independent of treatment assignment*.

Third, we assume that every treatment has a chance of being seen:

**Assumption 3** (Positivity). *Pr*(*T* = *t*) > 0 *for all t* ∈ T. *We assume that all treatments have some probability of being seen.*

The second and third assumptions are guaranteed by proper randomization of the experiment, whereas the first is generally understood to mean that there is no interference between units and no hidden values of treatment. For each observation, we observe only a single potential outcome corresponding to the realized treatment.

Building off this notation, we can introduce mathematical notation to cover high-dimensional text and the low-dimensional representation of texts derived from *g*, which we will use for our inferences. We start by extending our notation to cover multidimensional outcomes, **Y***_i_*, and multidimensional treatments, **T***_i_*. We will suppose, for now, that we have already determined *g*, the codebook function. Recall that *g* is applicable regardless of whether the coding is done by a machine learning algorithm, a team of research assistants, or an expert with decades of experience.

We write the set of possible values for the mapped text as 𝒵 with a subscript to indicate whether it is the dependent variable or treatment. We denote the realized values of the low-dimensional representation for unit *i* as **z***_i_* [*i* ∈ (1, …, *N*)]. We suppose that when the outcome is text *g*:𝒴 → 𝒵*_Y_* and **z***_i_* ≡ *g*(**Y***_i_*), and when the treatment is text *g*:T → 𝒵*_T_* and **z***_i_* ≡ *g*(**T***_i_*). The set 𝒵 is a lower-dimensional representation of the text and can take on a variety of forms depending on the study of interest. For example, if we are hand-coding our documents into two mutually exclusive and exhaustive categories, then 𝒵 ≡ {0,1}. If we are using a mixed-membership topic model to measure the prevalence of *K* topics as our dependent variable, then 𝒵 is a *K* − 1 dimensional simplex. In addition, if we are using texts as a treatment, we might suppose that 𝒵 is the set of *K* binary feature vectors, representing the presence or absence of an underlying treatment (although our workflow is general, we prefer binary treatments primarily for simplicity of functional form; see Supplement S7 for more). There are numerous other types of *g* that we might use—including latent scales, dictionary-based counts of terms, or crowd-sourced measures of content. While we generally assume that *g* substantially reduces dimensionality, the only requirement for *g* is that it is a function.

We next use *g* to write our causal quantity of interest in terms of the low-dimensional representation. To make this concrete, consider a case where we have a binary nontext treatment and a text-based outcome (we consider other causal estimands below). Suppose we hand-code each document into one of *K* categories such that for unit *i*, we can write the coded text under treatment as **z**_**i**_(1) ≡ *g*(**Y***_i_*(1)). We can then define the ATE for category *k* to be

$$\begin{array}{c} {\text{ATE}}_{k}=E[g{\left(Y_{i}(1)\right)}_{k}-g{\left(Y_{i}(0)\right)}_{k}]\\ =E[z_{i,k}(1)-z_{i,k}(0)] \end{array}$$ (1)

where *z*_*i*,*k*_(1) and *z*_*i*,*k*_(0) indicate the values of the *k*th category, for unit *i*, under treatment and control, respectively.

#### The problems: Identification and overfitting

Equation 1 supposes that we already have a *g* in hand. Whether by reading or machine learning, *g* is often discovered by interacting with some of the data. We denote the set of documents considered in development of *g* as **J** and write *g*_**J**_ to indicate the dependence of *g* on the documents. Problems of identification and estimation arise where the set of documents used to develop *g*, **J**, overlaps with the set of documents used in estimation, which we will call **I**. There are two broad concerns: an identification problem arising from FPCILV and an estimation problem with overfitting.

If Assumption 1 (SUTVA) holds, then each observation’s response does not depend on other units’ treatment status. However, even when Assumption 1 holds, when we discover *g*_**J**_, the particular randomization that we obtain will affect the *g*_**J**_ that we estimate. This dependence on a randomization occurs because the treatment vector **T**_**J**_—the treatment assignments for all documents **J**—affects the *g* that we obtain. If we were to randomize again, we would obtain a different **T**_**J**_ and therefore a different *g*_**J**_—a distinctively challenging form of data-adaptive estimation as in Hubbard *et al.* (*24*). This makes it impossible to compare the estimates across different randomizations because the content or even the number of categories might be different. Because the estimates cannot be compared, it is impossible to even define the bias, variance, consistency, or other properties of an estimator. Supplement S2 provides a formal definition of the FPCILV.

To see how the FPCILV works in practice, consider a stylized experiment on four units with a dichotomous intervention (treatment/control) and a text-based outcome. We might imagine potential outcomes that have a simple relationship between treatment and the text-based outcome such as the one shown in Table 1.

**Table 1.** A stylized experiment with text-based outcomes. (**A**) shows the potential outcomes for each unit under each treatment assignment. Treated units talk about candidate morals and polarization and control units talk about taxes and immigration. In (**B**), *T* denotes a different treatment assignment vector where two of four units are treated. *Y* denotes text-based observed outcomes under each treatment assignment. Mo, Im, Tx, and Po stand for candidate morals, immigration, taxes, and polarization, respectively.

(**A**) Text-based potential outcomes																
	**Potential outcome under treatment**			**Potential outcome under control**		
Person 1	Candidate morals			Taxes		
Person 2	Candidate morals			Taxes		
Person 3	Polarization			Immigration		
Person 4	Polarization			Immigration		
(**B**) Text-based observed outcomes under six different treatment assignments						
	T Y	T Y	T Y	T Y	T Y	T Y
Person 1	1 Mo	1 Mo	1 Mo	0 Tx	0 Tx	0 Tx
Person 2	1 Mo	0 Tx	0 Tx	1 Mo	1 Mo	0 Tx
Person 3	0 Im	1 Po	0 Im	1 Po	0 Im	1 Po
Person 4	0 Im	0 Im	1 Po	0 Im	1 Po	1 Po
Number of categories	2	4	4	4	4	2

Using Table 1, we can imagine the properties of an estimator applied to this text-based experiment as we rerandomize treatment assignment. Suppose that for each randomization we choose a *g* that measures each category we observe using an indicator variable and then, given *g*, estimate the treatment effect. For example, consider if we observe the treatment vector (1,1,0,0), we only observe two of the four categories: morals and immigration. Accordingly, our learned *g* consists of two indicator variables, one for morals and one for immigration. We could randomize again and get assignments (1,0,1,0), where we would observe all four categories resulting in four indicator variables. Under the randomization (0,0,1,1) (i.e., the sixth scenario in Table 1B), we are back to only two categories, taxes and polarization, and, thus, only two indicator variables.

As we randomize, we estimate different *g*’s with different categories. This lack of category stability complicates our ability to analyze our estimators as we traditionally do, using a workflow based on rerandomization. We take this category and classification stability for granted in standard experiments because categories are defined and fixed before the experiment. However, when we estimate categories from data, the discovered *g* depends on the randomization and thus induces dependence of a unit’s coded outcome on the treatment assignments of other units. Even if we fix the categories, as we might do with a supervised model, different randomizations may lead to different rules for assigning documents to categories, leading to a lack of classification stability. If, however, we fix *g* before estimating the effects, the problem is resolved, and the properties of the estimator are now well defined.

Even if we assume away the FPCILV, estimating *g* means that researchers might overfit: discover effects that are present in a particular sample but not in the population. The overfitting problem is particularly acute when a researcher is fishing—searching over *g*’s to obtain statistical significance or estimates that satisfy a related criterion. However, overfitting can occur even if researchers are conducting data analysis without ill intentions. Researchers necessarily search over different *g*’s to find those that best meet the criteria of interpretability, interest, fidelity, and tractability. Hand-coding requires iteration to refine the codebook, supervised models to refine the classifier, and unsupervised methods to adjust parameters and examine different organizations.

Fishing and overfitting are a problem in all experimental designs, not just those with text. The problem of respecifying *g* until finding a statistically significant result is analogous to the problem of researchers recoding variables or ignoring conditions in an experiment, which can lead to false-positive results (*25*). The problem with text-based inferences is heightened because texts are much more flexible than other types of variables, creating a much wider range of potential *g*’s. This wider range increases the risk of overfitting, even among well-intentioned analysts. Overfitting is also likely in texts because it is so easy to justify a particular *g* after the fact—the human brain is well equipped to identify and justify a pattern in a low-dimensional representation of text, even if that pattern emerges merely out of randomness. This means that validation steps alone may be an insufficient safeguard against overfitting, although texts provide a rich set of material to validate the content.

### A train/test split procedure for valid causal inference with text

To address the identification issues caused by the FPCILV and the estimation challenges of overfitting, we must break the dependence between the discovery of *g* and the estimation of the causal effect. The most straightforward approach is to define *g* before looking at the documents. Defining the categories beforehand, however, limits our coding scheme, excluding information about the language used in the experiment’s interventions or what units said in response to a treatment. If we define our codebook before seeing text, we will miss important concepts and have a poorer measure of key theoretical concepts.

We could also assume the problem away. Specifically, to eliminate the FPCILV, it is sufficient to assume that the codebook that we obtain is invariant to randomization. Take, for example, the text as outcome case; if the *g* we learned does not change over different randomizations of the treatment, we do not have an FPCILV. We define a formal version of this assumption in Supplement S2.

Our preferred procedure is to explicitly separate the creation of *g* and the estimation of treatment effects. This procedure avoids the FPCILV and provides a natural check against overfitting. To explicitly separate the creation of the codebook and its application to estimate effects, we randomly divide our data into a training set and a test set. Specifically, we randomly create a set of units in a training set denoted by the indices **J** and a nonoverlapping test set denoted by the indices **I**. We use only the training set to estimate the *g*_**J**_ function and then discard it. We then use the test set exclusively to estimate the causal effect on the documents in **I**.

This division between the training and test set addresses both the identification and estimation problems. It avoids the FPCILV in the test set because the function *g* does not depend on the randomization in the test set, so that each test set unit’s response depends only on its assigned treatment status. There is still a dependence on the training set observations and their treatment assignment. This, however, is analogous to the analyst shaping the object of inquiry or creating a codebook after a pretest. With the FPCILV addressed, it is now possible to define key properties of the estimator, like bias or consistency.

The sample split also addresses the concerns about overfitting. The analyst can explore in the training set as much as she likes, but, because findings are verified in a test set that is only accessed once, she is incentivized to find a robust underlying pattern. Patterns in the training set that are due to idiosyncratic noise are highly unlikely to also arise in the test set, which helps assure the analyst that patterns that are confirmed by the separate test set will be replicable in further experiments. By locking *g* into place in the training set, the properties of the tests in the test set do not depend on the number of different *g*’s considered in the training set. In practice, we find that splitting the sample ensures that we are able to consider several models to find the *g* that best captures the data and aligns with our theoretical quantity of interest without worrying about accidentally p-hacking.

With the reason for sample splitting established, we first describe our final estimands for the text as outcome and text as treatment cases. We then describe the pragmatic steps we suggest to implement a train/test split. Then, we discuss the trade-offs in using a split-sample approach. Having described our strategy, we connect our approach to existing prior work before demonstrating how it works in two different applications.

#### Text as outcome

In text as outcome, the particular *g* that the analyst chooses defines the categories of the outcome from which the estimand will be defined. Our goal is to obtain a consistent (and preferably unbiased) estimator for the ATE (or other causal quantities of interest) assuming a particular *g*. Using Assumptions 1 to 3, the following is a consistent estimator

$$\hat{\mathrm{ATE}}=\sum_{i\in I}\frac{I(T_{i}=1)g_{J}(Y_{i})}{\sum_{i\in I}I(T_{i}=1)}-\sum_{i\in I}\frac{I(T_{i}=0)g_{J}(Y_{i})}{\sum_{i\in I}I(T_{i}=0)}$$

Supplement S2 gives an identification proof. The proof relies on the fact that *g* is fixed before documents **I** are examined, which allows us to treat the mapped outcome *g*_**J**_(**Y**_**I**_) as an observed variable.

#### Text as treatment

Text may also be the treatment in an experiment (*26*, *27*). For example, we may ask individuals to read a candidate’s biography and then evaluate how the candidate’s favorability on a scale of 0 to 100 (*17*). The treatment, **T***_i_*, is the text description of the candidate assigned to the respondents. The potential outcomes *Y_i_*(**T***_i_*) describes respondent *i*’s rating of the candidate under the treatment assigned to respondent *i*.

While we could compare two completely separate candidate descriptions as in A/B tests, social scientists are almost always interested in how some underlying feature of a document affects responses. That is, the researcher is interested in estimating how an aspect or latent value of the text influences the outcome, as in Voelkel *et al.* (*27*). For example, the researcher might be interested in whether including military service in the description has an impact on the respondents’ ratings of the candidate. Military service is a latent variable—there are many ways that the text could describe military service that all would count as the inclusion of military service and many ways that the text could omit military service that all would count as the absence of the latent variable. The researcher might assign 100 different candidate descriptions, some of which mention the candidate’s military service and some of which do not. In this case, the treatment of interest is *Z_i_* ≡ *g*(**T***_i_*) ∈ {0,1}, which maps the treatment text to an indicator variable that indicates whether the text contains a description of the candidate’s military service. To estimate the impact of a binary treatment, we could use the estimator

$$\hat{\mathrm{ATE}}=\sum_{i\in I}\frac{I(Z_{i}=1)Y_{i}}{\sum_{i\in I}I(Z_{i}=1)}-\sum_{i\in I}\frac{I(Z_{i}=0)Y_{i}}{\sum_{i\in I}I(Z_{i}=0)}$$

where *Z_i_* ≡ *g*_**J**_(**T***_i_*).

With text as treatment, we may be interested in more than just one latent treatment. The presence of multiple latent treatments requires different causal estimands and enables us to ask different questions about how features of the text affect responses. For example, we can learn the marginal effect of military service and how military service interacts with other features of the candidate’s background—such as occupation or family life. Typically with multidimensional treatments, we are interested in the effect of one treatment holding all others constant. This complicates the use of topic models that suppose 𝒵 is a simplex (all topic proportions are nonnegative and sum to one) because there is no straightforward way to change one topic holding others constant [see (*17*) and Supplement S7]. Instead, we will work with *g* that compresses the text **T** to a vector of *K* binary measured treatments **Z** ∈ 𝒵, where 𝒵 represents all 2*^K^* possible combinations of the measured treatments, and **Z** has typical element *Z_ik_*, indicating the *k*th treatment for observation *i*. We could also, of course, suppose that *g* maps **T** to a set of continuous underlying treatments, but this requires additional functional form assumptions. Fong and Grimmer (*28*) also suppose that there are a series of unmeasured treatments **B***_i_* ∈ ℬ, which we obtain by applying the function *h* to the texts so that *h*(**T***_i_*) ≡ **B***_i_*. Fong and Grimmer (*28*) then suppose that the combination of measured and unmeasured treatments captures all the relevant features of the text or that *Y_i_*(**T***_i_*) = *Y_i_*(*g*(**T***_i_*), *h*(**T***_i_*)) = *Y_i_*(**z***_i_*, **B***_i_*).

If **Z** ∈ {0,1}, then we can define the ATE as

$$\text{ATE}=\sum_{b\in B}E[Y_{i}(Z_{i}=1,B_{i}=b)-Y_{i}(Z_{i}=0,B_{i}=b)]\text{Pr}(B_{i}=b)$$

If **Z** is higher-dimensional, we can generalize this estimand to be the average marginal component effect.

To estimate the effect of measured latent treatments, we require an additional assumption than in the text as outcome case. This is because we are usually only able to randomize at the text level, but we are interested in identifying the effect of latent treatments we are unable to manipulate directly, raising the possibility that our measured treatment of interest **Z***_i_* could be confounded by **B***_i_*. Consequently, we need to make an additional assumption beyond the three mentioned above [SUTVA, Ignorability and Positivity, which, in the multidimensional case, generalizes to *f*(**Z***_i_*) > 0 for all $Z_{i}\in \text{Range}\;g(\cdot )$]. Specifically, Fong and Grimmer (*28*) show that one of the following two assumptions is sufficient to identify the ATE and to ensure that the difference in means estimator is a consistent estimator.

**Assumption 4.** (*28*) *Either*

*1) The measured and unmeasured latent treatments are independent Pr*(**Z***_i_*, **B***_i_*) *= Pr*(**Z***_i_*)*Pr*(**B***_i_*) *or*

*2) The unmeasured treatments have no effect on the outcome: E*[*Y_i_*(*Z_i_* = *z*, **B***_i_* = **b**)] = *E*[*Y_i_*(*Z_i_* = *z*, **B***_i_* = **b**′)] *for all z* ∈ {0,1} *and all* **b**, **b**′ ∈ ℬ

Fong and Grimmer (*28*) show that Assumptions 4.1 and 4.2 are analogous to assumptions made when doing standard observational causal inference work. Assumption 4.1 implies that any omitted unmeasured treatments are not systematically related to the measured treatments of interest and therefore cannot confound our estimate. Assumption 4.2 implies that any unmeasured treatment cannot affect the outcome, and as a result, it cannot confound our estimate. Since the assumptions depend on the distribution of latent components of the text in the population, they are not guaranteed by randomization at the level of the complete texts. Fong and Grimmer (*28*) provide a set of tools for diagnosing whether the assumptions hold for potentially confounding unmeasured treatments.

#### Procedure

In this section, we discuss the general procedure for implementing the train/test split to estimate the above quantities of interest. This procedure follows the schematic in Fig. 1. Considerations specific to text-as-treatment or text-as-outcome are deferred to Supplements S6 and S7.

##### Step 1: Splitting the sample.

The first major choice that the analyst faces is how to split the sample into two pieces: the training set and the test set. A default recommendation is to split 50% of the documents in training and 50% in the test set. However, this depends on how the researcher evaluates the trade-off between discovery of *g* and testing. Additional documents in the training set enables learning a more complicated *g* or more precise coding rules. Additional documents in the test set enable more precise estimation of the treatment effect. While the test set should be representative of the population that you want to make inference about, the training set can draw on additional nonrepresentative documents as long as they are similar enough to the test set to aid in learning a useful *g*. Last, when taking the sample, the analyst can stratify on characteristics of interest to ensure that the split has appropriate balance between the training and test set on those characteristics.

Once the test set is decided, the single most important rule is that the test set is used once, solely for estimation. If the analyst revises *g* after looking at the test set data, she reintroduces the FPCILV and risks overfitting. Test data must be set aside before any part of the analysis: Even preliminary steps like preprocessing must not include the test dataset. Third parties, such as survey firms and research agencies, can be helpful in credibly setting the data aside.

##### Step 2: Discover g.

We use the training set and text as data methods to find a *g* that is interpretable, is of theoretical interest, has high label fidelity, and is tractable. Here, we use the Structural Topic Model (STM) and the Supervised Indian Buffet Process (sIBP), but there are numerous other methods that are applicable.

##### Step 3: Validation in the training set.

Validation is an important part of the text analysis process, and researchers should apply the normal process of validation to establish label fidelity. These validations are often application specific and draw on close reading of the texts. These validations should be completed in the training set as part of the process of discovering and labeling *g* before the test set is opened. See Grimmer and Stewart (*29*) for more details on types of validation and the STM package (*30*) for tools designed to assist with validation.

During this step, we can refit *g* as often as it is useful for our analysis. However, once applied to the test set, we cannot alter *g* further. Before fixing *g*, ensure that *g* is capturing the aspect of the texts that you want to capture, assign labels, and then validate to ensure that the conceptual gap between those labels and the representation *g* produces is as small as possible. While validation approaches may vary—this necessarily involves reading documents (*15*, *22*, *29*)—it is helpful to fix a set of human-assigned labels, example documents, and automated keyword labels in advance to avoid subtle influence from the test set.

In addition, while we focus on inference challenges with *g*, standard experimental challenges remain. We advise analysts to fix their evaluation plans before looking at data in the test set. Here, we can draw from the established literature on best practices in experiments potentially including a pre-analysis plan (*31*). This can include multiple-testing and false-discovery rate corrections.

##### Step 4: Applying g and estimating causal effects.

Mechanically, applying *g* in the test set is straightforward and is essentially the process of making a prediction for an unseen document. After calculating the quantities *g*_**J**_(**Y**_**I**_), we can use standard estimators appropriate to our estimand, such as the difference of means, to estimate the ATE. The supplement describes how to apply *g* to test documents in both the sIBP and the STM, which we cover in our examples.

##### Step 5: Validation in the test set.

It is also necessary to ensure that the model fits nearly as well on the test set as it did on the training set. When both the training and test sets are random draws from the same population, this will generally be true, but overfitting is still possible, particularly with small sample sizes. The techniques used to validate the original model can be used in the test set as well as common measures of model fit such as log likelihood. Unlike the validation in the training set, during the validation in the test set, the analyst cannot return to make changes to the model. Nevertheless, validation in the test set helps the analyst understand the substantive meaning of what is being estimated and provides guidance for future experiments. Formally, our estimand is defined in terms of the empirically discovered *g* in the training set. However, invariably, the analyst makes a broader argument indicated by the label. Validation in the test set verifies that label fidelity holds and that *g* represents the concept in the test set of documents.

#### Trade-offs

The train/test split addresses many of our concerns, but it is not without cost. Efficiency loss is the biggest concern. In a 50/50 train-test split, half the data are used in each phase of analysis, implying that half the data are excluded from each step. At the outset, it is difficult to assess how much data are necessary for either the training or the test set. The challenge in setting the size of the test set is that the analyst does not yet know what the outcome (or treatment) will be when the decision is made on the size of the split. The problem in setting the size of the training set is that we do not know the power we need for discovery. Alternatively, we could focus first on determining the power needed for estimation of an effect and then allocate the remaining data for discovery. This can be effective, but it requires that we are able to anticipate characteristics of a treatment or outcome we have not yet discovered.

Another concern is conceptual: We may worry that the particular *g*, and perhaps even the ultimate conceptualization, might depend on how the sample was originally split. While this is an important issue, we note that it is a common feature across research: Our ideas and views of the world will be informed by the order of evidence that we encounter. Two different teams—or even the same team on different days—might look at the same data and find different things of salience to measure. This does not invalidate any one reading of the text, but it does require that analysts and readers keep in mind that this is only one possible interpretation of the events in the world. This is true even in classical nontext randomized experiments. Consider a job training experiment where we could choose to measure any of a wide variety of outcomes from employment, wages, social connections, or even mental well-being. That one of these outcomes shows no effect would not be to suggest that the job training had no effect on the people involved, just that it did not have an effect on that specific outcome. Furthermore, although two teams might pick two different ways of measuring employment, their findings are likely to coincide because they are looking at the same underlying fact pattern.

It is essential to ask how well *g* fits on test data, whether it measures the concept it intends to well, and to explore whether there are critical concepts emerging or missing from the analysis. It is by validating *g* that we ensure that results we find will be consistent with other measurements of the same concept. Just like other approaches to research, as *g*’s are applied to different datasets and interpreted by different researchers, our hope is that the particular data we are using will matter less than the broader truth the analysis reveals.

#### Prior work

Our central contribution is a rigorous machine learning workflow that characterizes how to make causal inferences with texts and identifies problems that arise when making those causal inferences and the explanation of why sample splitting addresses these challenges. Together, this serves as a unified guidebook for using text and causal inference. Before Roberts *et al.* (*32*) and Fong and Grimmer (*17*), there had been comparatively little work on causal inference with latent variables. Lanza *et al.* (*33*) consider causal inference for latent class models but do not give a formal statement of identifying assumptions or acknowledge the set of concerns we identify as the FPCILV. Volfovsky *et al.* (*34*) present a variety of estimands and estimation strategies for causal effects where the dependent variable is ordinal. They provide approaches based on both the observed data and latent continuous outcomes. Volfovsky *et al.* (*34*) express caution about the latent variable formulation due to identification concerns, and the subsequent literature (e.g., *35*) has moved away from it. Unfortunately, many of their strategies based directly on the observed outcomes are unavailable in the much higher-dimensional setting of text analysis. Following our posting of the preprint version of this article in 2018, there has been a burst of interest from the natural language processing community exploring various components of causal inference with text classifiers and language models (*36*–*38*).

Our proposed solution, sample splitting, has a long history in machine learning. There has been a growing exploration of the use of train/test splits in the social sciences as well as causal inference (*19*, *21*, *39*). It is the natural solution to this class of problems, and we certainly do not claim to be the first to introduce the idea of train/test splits into the area. Our approach is mostly closely related to prior work by Fafchamps and Labonne (*20*) and Anderson and Magruder (*39*), which both advocate a form of split samples to aid in discovery with standard regression analysis.

Our work is also part of a burgeoning literature on the use of machine learning algorithms to enhance causal inference (*18*, *19*, *21*, *40*–*43*). Much of this work focuses on estimating causal parameters on observed data and addressing a common set of concerns, such as estimation and inference in high-dimensional settings, regularization bias, and overfitting. Our work complements this literature by exploring the use of latent treatments and outcomes. Many pieces in this area call for sample splits or cross-validation for estimation and inference, providing additional justification for our preferred approach [e.g., (*21*)]. In Supplement S3, we discuss the connection between our work and related work using cross-validation.

### Applications

Our preferred approach is sequential. We advocate a split-sample design that allows for a discovery phase to precede the analysis phase. We also explicitly plan to run experiments again, using each analysis to inform future work. In this section, we demonstrate this approach through two applications: one where text is the outcome and one where text is the treatment. In each case, we explicitly describe the discovery process. Although we use specific models to facilitate discovery, STM for text as outcome and sIBP for text as treatment, the process we describe here is general to any method for discovering *g* from data.

#### Text as outcome: An experiment on immigration

To first demonstrate how to use text as a response in a causal inference workflow, we apply the STM to open-ended responses from a survey experiment on immigration (*32*). Specifically, we build on an experiment first introduced in Cohen *et al.* (*44*) to assess how knowledge about an individual’s criminal history affects respondent’s preference for punishment and deportation. These experimental results contribute to a large literature about Americans’ preferences about immigrants and immigration policy [see (*45*) for a review] and a literature on the punishments people view as appropriate for crimes. Critically, in both conditions of our experiment, an individual has broken the same law, entering the country illegally, but differs solely on prior criminal history. We therefore ask how someone’s past criminal behavior affects the public’s preference for future punishment and use the open-ended responses to gather a stated reason for that preference.

We analyze three iterations of a similar experiment. With each experiment, we chose *g* and estimated treatment effects using the process described in Fig. 1. The first results are based on responses initially using the data from Cohen *et al.* (*44*). We use this initial set of responses to estimate an initial *g* and to provide baseline categories for the considerations respondents raise when explaining why someone deserves punishment. In a second experiment, we build on (*44*), but address issues in the wording of questions, expand the set of respondents who are asked to provide an open-ended response, and update the results with contemporary data. We then run a third experiment because we found that our *g* performed poorly in the test set of the second experiment. We also used that opportunity to improve small features of the design of the experiment. We describe each experiment in detail below.

We report the results of experiments 1 and 3 (rather than just 3) in the supplement and provide the data for all three experiments in our replication archive to be transparent about our research process, something we suggest that researchers do to avoid selective reporting based on an experiment’s results. The experimental results show that there has been unexpected stability in the considerations Americans raise when explaining their punishment preferences, although there are some additional categories that emerge. There is also a consistent inclination to punish individuals who have previously committed a crime, although they committed the same crime as someone without a criminal history.

In each experiment, we used equal proportions of the sample in the train and test sets. In each experiment, we fit several models in the training set before choosing a single model that we then applied to the test set.

For experiment 3, Table 2 shows the words with the highest probability in each of the 11 topics. Topics range from advocating for rehabilitation or assistance for remaining in the country to suggesting that the person should receive maximal punishment. Because of space constraints, we put additional details about the other experiments in Supplement S8.

**Table 2.** Topics and highest probability words for experiment 3.

	**Label**	**Highest probability** **words**
Topic 1	Limited punishment with help to stay in country, complaints about immigration system	legal, way, immigr, danger, peopl, allow, come, countri, can, enter
Topic 2	Deport	deport, think, prison, crime, alreadi, imprison, illeg, sinc, serv, time
Topic 3	Deport because of money	just, send, back, countri, jail, come, prison, let, harm, money
Topic 4	Depends on the circumstances	first, countri, time, came, jail, man, think, reason, govern, put
Topic 5	More information needed	state, unit, prison, crime, immigr, illeg, take, crimin, simpli, put
Topic 6	Crime, small amount of jail time, then deportation	enter, countri, illeg, person, jail, deport, time, proper, imprison, determin
Topic 7	Punish to full extent of the law	crime, violent, person, law, convict, commit, deport, illeg, punish, offend
Topic 8	Allow to stay, no prison, rehabilitate, probably another explanation	dont, crimin, think, tri, hes, offens, better, case, know, make
Topic 9	No prison, deportation	deport, prison, will, person, countri, man, illeg, serv, time, sentenc
Topic 10	Should be sent back	sent, back, countri, prison, home, think, pay, origin, illeg, time
Topic 11	Repeat offender, danger to society	believ, countri, violat, offend, person, law, deport, prison, citizen, individu

**Fig. 2.**
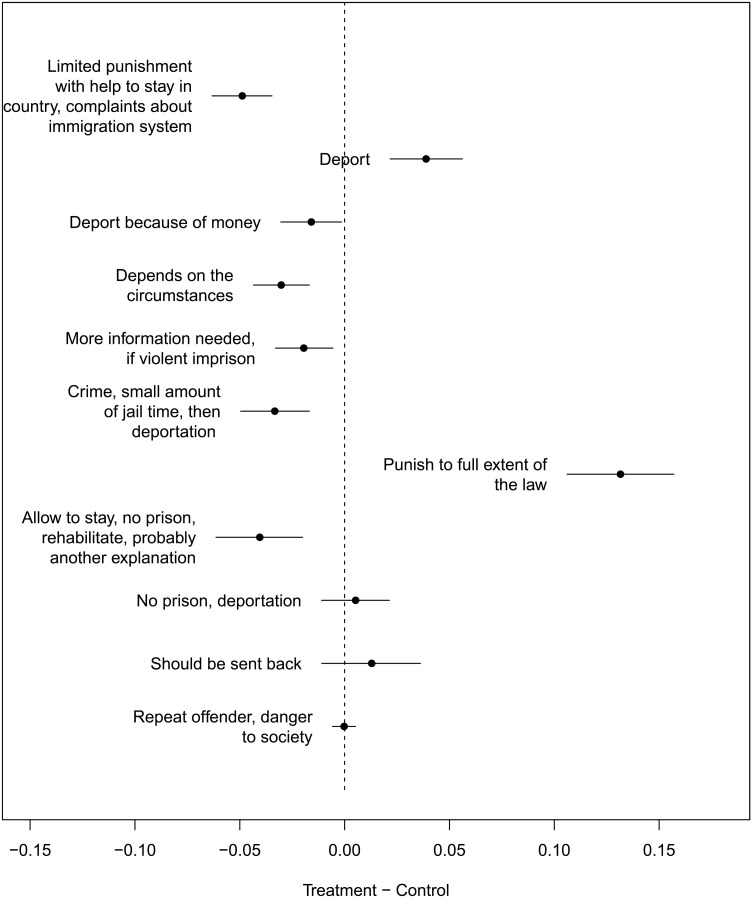
Test set results for immigration experiment 3. Point estimates and 95% confidence intervals.

After discovering, labeling, and finalizing *g* in the training set, we estimated the effect of treatment on the topics in the test set. In Fig. 2, we show large impacts of treatment on topics. Treatment (indicating that the person had a previous criminal history) increased the amount of writing about maximal punishment, deportation, and sending the person back to their country of origin. The control group was more likely to advocate that the person should be able to stay in the country or that the punishment should depend on the circumstances of the crime. We found qualitatively similar results in our other experiments (Supplement S8), although *g* is different in both cases and the set of people who were asked to provide a reason is different. In each case, the description of a criminal history substantially increases the likelihood that the respondent advocates for more severe punishment or deportation.

In Fig. 1, we recommend concluding experiments with suggestions for further experimentation, and we do so here. Future iterations of the experiment could explore two features of the treatment. First, we have only provided information about one type of crime. It would be revealing to know how individuals respond to crimes of differing severity. Second, we could use our existing design to estimate heterogeneous treatment effects, which would be particularly interesting in light of contemporary debates about how to handle undocumented immigration in the United States.

#### Text as treatment: CFPB

We turn next to apply our workflow to text-based treatments. We examine the features of a complaint that cause the Consumer Financial Protection Bureau (CFPB) to reach a timely resolution of the issue.

Our goal is to discover the treatments and estimate their effect on the probability of a response. We discover *g* using the sIBP developed for this setting in Fong and Grimmer (*17*) and implemented in the texteffect package in R (*46*). The model learns a set of latent binary features that are predictive of both the text and the outcome. To do this, we first randomly divide the data, placing 10% in the training set and 90% of the data in the test set. We place more data in the test set because our large sample (≈11K) provides ample opportunity to discover the latent treatments in the training set and to provide greater power when estimating effects in the test set. In the training set, we apply the sIBP to the text of the complaints and whether there was a timely response. We use an extensive search to determine the number of features to include and the particular model run to use. Materials and Methods provides more details on the sIBP.

Once we have fit the model in the training set, we use it to infer the treatments in the test set. Table 3 provides the inferred latent treatments from the CFPB complaint data. The Automatic Keywords are the words with the largest values in the estimated latent factors for each treatment, and the manual keyword is a phrase that we assign to each category after assessing the categories. Using these features, we can then infer their presence or absence in the treated documents and then estimate their effect. To do this, we use the regression procedure from Fong and Grimmer (*17*) and then use a bootstrap to capture uncertainty from estimation.

**Table 3.** CFPB latent treatments

**No.**	**Automatic keywords**	**Manual keyword**
1	mortgage, loan, payments, modification, foreclosure, property	Mortgage
2	call, called, told, asked, hung, number	Narrative
3	credit_report, disputed, credit_ reporting, fcra, reporting, report	Credit score
4	xxxx, xxxx_xxxx, letter, request, documents, time	Detailed
5	account, payment, xxxx, balance, credit, card	Credit card

**Fig. 3.**
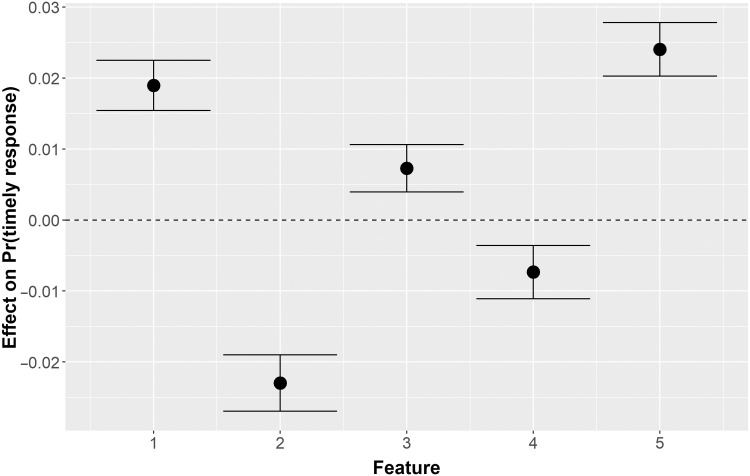
The effect of complaint features on a prompt response.

Figure 3 shows the effects of each latent feature on the probability of a timely response. The black dots are point estimates, and the lines are 95% confidence intervals. Figure 3 reveals that when consumers focus on the specific banking activities of home mortgages (treatment 1), credit scores (treatment 3), and personal banking (treatment 5), the probability of a prompt response increases. In contrast, the CFPB is much less successful at obtaining prompt responses given the narration of the event (treatment 2) or detailed documentation (treatment 4). Presumably, the former sorts of complaints trigger programmatic considerations that can be resolved by reference to the relevant policy, while the latter must be dealt with by banks on a case-by-case basis and therefore require more time.

If we were to run a further iteration of the CFBP analysis, we would proceed on two fronts. First, there is a constant stream of data arriving at the CFPB. We could use our existing *g* to reestimate the treatment effects to see whether there are temporal trends. We could also estimate a different *g* to assess whether categories emerge over time. Second, we could design experiments to address concerns about demographic differences. For example, we could partner with individuals who are planning to write complaints to see how their language, independent of their personal characteristics, affects the response.

## DISCUSSION

Text is inherently high-dimensional. This complexity makes it difficult to work with text as an intervention or an outcome without some simplifying low-dimensional representation. There are a whole host of methods in the text as data toolkit for learning undiscovered, insightful representations of text data. Unfortunately, while these low-dimensional representations make text comprehensible at scale, they also make causal inference with text difficult to do well, even within an experimental context. When we discover the mapping between the data and the quantities of interest, the process of discovery undermines the researcher’s ability to make credible causal inference.

Here, we have introduced a rigorous machine learning workflow for causal inference with text, identified problems that emerge when using text data for causal inference, and then described a procedure to resolve those problems. In this conceptual framework, we have clarified the central role of *g*, the codebook function, in making the link between the high-dimensional text and our low-dimensional representation of the treatment or outcome. In doing so, we clarify two threats to causal inference: the FPCILV—an identification issue—and overfitting—an estimation issue. We demonstrate that both the identification and estimation concerns can be addressed with a simple split of the dataset into a training set used for discovery of *g* and a test set used for estimation of the causal effect. More broadly, we advocate for research designs that allow for sequential experiments that explicitly set aside research degrees of freedom for discovery of interesting measures, while rigorously testing relationships within experiments once these measures are defined explicitly.

Our workflow unifies the text as data literature with the traditional approaches to causal inference. We have considered the text as treatment and text as outcome, and in the future, we hope to address the setting where text is both treatment and outcome. In related work, Roberts *et al.* (*47*) introduce techniques for matching on text to adjust for confounders, and recent papers (*48*, *49*) consider other text-based confounding approaches. There is much more work to be done to explore other causal designs, causal effects in conversations (*50*), optimally setting training/test splits, and increasing the efficiency of discovery methods so that they can work on even smaller datasets.

While our argument has principally been about the analysis of text data, our work has implications for any latent representation of a treatment or outcome used when making a causal inference. This could include latent measures common in social science, such as measures of democracy (e.g. polity), voting behavior (e.g. ideal points), and forms of manual content analysis. Any time a process of discovery is necessary, we should be concerned if the discovery is completed on the same units where the effect is estimated. In certain circumstances, this process will be unavoidable. Polity scores were developed by looking at the full population of world democracies; thus, there is no test set we can access, but we argue that the train/test split should be considered in the context of the development of future measures that require a low-dimensional representation of high-dimensional data.

What do our findings mean for existing applied work (text and otherwise)? The FPCILV and overfitting raise considerable risks to replicability, but it does not mean that any work not using a train/test split is invalid. However, as estimands based on latent constructs become more common in the social sciences, we hope to see an increased use of the train/test split and the development of creative methodologies to enhance the process of discovery.

## MATERIALS AND METHODS

### Text as outcome: An experiment on immigration

#### Experiment 1

As a starting point, we conduct an analysis of the experimental results reported in (*44*). The survey experiment was administered in the context of a larger study of public perceptions of the criminal justice system. The survey was conducted in 2000 by telephone random-digit dial and includes 1300 respondents. More details about the survey are available in Cohen *et al.* (*51*).

In the experiment, respondents were given two scenarios of a criminal offense. In both the treatment and control conditions, the same crime was committed: illegal entry to the United States. In the treatment condition, respondents were told that the person had previously committed a violent crime and had been deported. In the control condition, respondents were told that the person had never been imprisoned before.

The treatment condition prompt was as follows:

“A 28-year-old single man, a citizen of another country, was convicted of illegally entering the United States. Prior to this offense, he had served two previous prison sentences each more than a year. One of these previous sentences was for a violent crime and he had been deported back to his home country.”

In the control condition, respondents were told the following:

“A 28-year-old single man, a citizen of another country, was convicted of illegally entering the United States. Prior to this offense, he had never been imprisoned before.”

Respondents were then asked a closed-ended question about whether the person should go to jail. If they responded that the person should not go to jail, they were asked to respond to an open-ended question, “Why?” The key inferential goal of the initial study was to determine whether a respondent believed a person should be deported, jailed, or given some other punishment.

#### Experiment 2

After analyzing the results of experiment 1, we ran a second experiment using the same treatment and control conditions but with slight design differences to build upon and improve the original experimental protocol. First, all respondents were asked the open-ended question, not just those who advocated for not sending the individual to jail. Second, we redesigned the survey to avoid order effects. Third, we asked a more specific open-ended question. We still asked “Should this offender be sent to prison?” (responses: yes, no, and do not know) but followed by asking “Why or why not? Please describe in **at least two sentences** what actions if any the U.S. government should take with respect to this person and why?” Per our Institutional Review Board, we added the statement “(Please **do not** include any identifying information such as your name or other information about you in this open-ended response.)” Experiment 2 was run on Mechanical Turk (MTurk) between 30 June 2017 and 16 July 2017 with 1299 respondents.

#### Experiment 3

We expected experiment 2 to be our last experiment, but we encountered a design problem. After we estimated *g* in the training set using STM and fit it to the test data, we realized that some of our topic labels were inaccurate. In particular, we had attempted to label topics using three predetermined categories: prison, deport, and allow to stay. However, the data in the test set suggested some additional categories. We could not simply relabel the topics in the test set because this would nullify the value of the train/test split. Instead, we decided to run an additional experiment. We also took the opportunity to make a few design changes. We had previously included an attention check, which appeared after the treatment question. We moved the attention check to before the treatment. We also had not previously used the MTurk qualification enforcing the location to be in the United States, although we did in experiment 3. Last, we blocked workers who had taken the survey in experiment 2 using the MTurkR package (*52*). We include the data from experiment 2 in our replication package but, because of poor topic labels, only present results from experiments 1 and 3 in the paper.

Experiment 3 was run on MTurk on 10 September 2017 with 1094 respondents. To avoid labeling mistakes, two members of our team labeled the topics independently using the training data and then compared labels with one another to create a final set of congruent labels before applying the *g* to the test set.

### Text as treatment: The CFPB

The CFPB is a product of Dodd-Frank legislation and is (in part) charged with offering protections to consumers. The CFPB solicits complaints from consumers across a variety of financial products and then addresses those complaints. It also has the power to secure payments for consumers from companies, impose fines on firms found to have acted illegally, or both.

The CFPB is particularly compelling for our analysis because it provides a massive database on the text of the complaint from the consumer and how the company responded. If the person filing the complaint consents, the CFPB posts the text of the complaint in their database, along with a variety of other data about the nature of the complaint. For example, one person filed a complaint stating that

“the service representative was harsh and not listening to my questions. Attempting to collect on a debt I thought was in a grace period ... They were aggressive and unwilling to hear it.”

and asked for remedy. The CFPB also records whether a business offers a timely response once the CFPB raises the complaint to the business. In total, we use a collection of 113,424 total complaints downloaded from the CFPB’s public website. Since we circulated the first draft of this paper in 2018, we have been pleased to see other researchers adopt this application as a test case for their own approaches to causal inference with texts.

The texts are not randomly assigned to the CFPB, but we view the use of CFPB data as still useful for demonstrating our workflow. Much of the information available to bureaucrats at the CFPB will be available in the complaint because of the way complaints are recorded in the CFPB data. To be clear, for the effect of the text to be identified, we would need to assume that the texts provide all the information for the outcome and that any remaining information is orthogonal to the latent features of the text. We view the example of the CFPB as useful because it provides us a clear way to think through how this assumption could be violated. If there are other nontextual factors that correlate with the text content, then our estimated treatment effects will be biased. For example, if working with the CFPB directly to resolve the complaint were important and individuals who submitted certain kinds of complaints were less well equipped to assist the CFPB, then we would be concerned about whether selection on observables holds, or there could be demographic factors that confound the analysis. For example, minorities may receive a slower response from CFPB bureaucrats or a more adversarial response from financial institutions (*53*), and minorities may be more likely to write about particular topics. While this is certainly plausible, many of the effects that we estimate of the text are large, so they would be difficult to explain solely through this confounding. Furthermore, Fong and Grimmer (*28*) demonstrate how to adjust for both text- and nontext-based confounders, such as the content of complaints or who submitted the complaint.

We use the sIBP to discover treatments in our corpus. The sIBP is a nonparametric Bayesian method; on the basis of a user-set hyperparameter, it estimates the number of features to include in the model, although the number estimated from a nonparametric method rarely corresponds to the optimal number for a particular application. To select a final model, we then evaluate the candidate model fits using a model fit statistic introduced in Fong and Grimmer (*17*). The train/test split ensures that we can refit the model several times, estimating the features that provide the best substantive insights.

## Supplementary Materials

### This PDF file includes:

Supplementary Texts Figs. S1 and S2 Tables S1 to S4 References

## References

[1] N. Gandhi, W. Zou, C. Meyer, S. Bhatia, L. Walasek, Computational methods for predicting and understanding food judgment. Psychol. Sci. 33, 579–594 (2022). 35298316 10.1177/09567976211043426

[2] S. Bhatia, C. Y. Olivola, N. Bhatia, A. Ameen, Predicting leadership perception with large-scale natural language data. Leadersh. Q. 101535 (2021).

[3] M. Bertrand, D. Karlan, S. Mullainathan, E. Shafir, J. Zinman, What’s advertising content worth? Evidence from a consumer credit marketing field experiment. Q. J. Econ. 125, 263–305 (2010).

[4] J. Berger, A. Humphreys, S. Ludwig, W. W. Moe, O. Netzer, D. A. Schweidel, Uniting the tribes: Using text for marketing insight. J. Mark. 84, 1–25 (2020).

[5] K. Myers, The elasticity of science. Am. Econ. J. Appl. Econ. 12 (4), 103–134 (2020).

[6] S. Bhatia, R. Richie, Transformer networks of human conceptual knowledge. PsyArXiv. 13 November 2020. 10.1037/rev0000319 36301272

[7] A. E. Boydstun, *Making the News: Politics, the Media, and Agenda Setting* (University of Chicago Press, 2013).

[8] A. Catalinac, From pork to policy: The rise of programmatic campaigning in Japanese elections. J. Polit. 78, 1–18 (2016).

[9] A. Spirling, U.S. treaty making with American Indians: Institutional change and relative power, 1784–1911. Am. J. Polit. Sci. 56, 84–97 (2012).

[10] D. B. Rubin, Estimating causal effects of treatments in randomized and nonrandomized studies. J. Educ. Psychol. 66, 688–701 (1974).

[11] J. Pearl, *Causality* (Cambridge Univ. Press, 2009).

[12] I. Lundberg, R. Johnson, B. M. Stewart, What is your estimand? Defining the target quantity connects statistical evidence to theory. Am. Sociol. Rev. 86, 532–565 (2021).

[13] M. Laver, K. Benoit, J. Garry, Extracting policy positions from political texts using words as data. Am. Polit. Sci. Rev. 97, 311–331 (2003).

[14] J. W. Pennebaker, M. R. Mehl, K. G. Niederhoffer, Psychological aspects of natural language use: Our words, our selves. Annu. Rev. Psychol. 54, 547–577 (2003). 12185209 10.1146/annurev.psych.54.101601.145041

[15] K. M. Quinn, B. L. Monroe, M. Colaresi, M. H. Crespin, D. R. Radev, How to analyze political attention with minimal assumptions and costs. Am. J. Polit. Sci. 54, 209–228 (2010).

[16] G. W. Imbens, D. B. Rubin. *Causal Inference in Statistics, Social, and Biomedical Sciences* (Cambridge Univ. Press, 2015).

[17] C. Fong, J. Grimmer, Discovery of Treatments from Text Corpora, in *Proceedings of the 54th Annual Meeting of the Association for Computational Linguistics* (Long Papers, 2016), vol. 1, pp. 1600–1609.

[18] J. Robins, L. Li, E. Tchetgen, A. van der Vaart, Higher order influence functions and minimax estimation of nonlinear functionals, in *Probability and Statistics: Essays in Honor of David A. Freedman* (Institute of Mathematical Statistics, 2008), pp. 335–421.

[19] S. Wager, S. Athey, Estimation and inference of heterogeneous treatment effects using random forests. J. Am. Stat. Assoc. 113, 1228–1242 (2018).

[20] M. Fafchamps, J. Labonne, Using split samples to improve inference on causal effects. Polit. Anal. 25, 465–482 (2017).

[21] V. Chernozhukov, D. Chetverikov, M. Demirer, E. Duflo, C. Hansen, W. Newey, J. Robins, Double/debiased machine learning for treatment and structural parameters. J. Econom. 21, C1–C68 (2018).

[22] K. Krippendorff, *Content Analysis: An Introduction to Its Methodology* (Sage, 2004).

[23] D. B. Rubin, Comment on “randomization analysis of experimental data: The fisher randomization test” by D. Basu. J. Am. Stat. Assoc. 75, 591–593 (1980).

[24] A. E. Hubbard, S. Kherad-Pajouh, M. J. van der Laan, Statistical inference for data adaptive target parameters. Int. J. Biostat. 12, 3–19 (2016). 27227715 10.1515/ijb-2015-0013

[25] J. P. Simmons, L. D. Nelson, U. Simonsohn, False-positive psychology: Undisclosed flexibility in data collection and analysis allows presenting anything as significant. Psychol. Sci. 22, 1359–1366 (2011). 22006061 10.1177/0956797611417632

[26] L. Vavreck, *The Message Matters* (Princeton Univ. Press, 2009).

[27] J. G. Voelkel, M. Malik, C. Redekopp, R. Willer, “Changing Americans’ attitudes about immigration: Using moral framing to bolster factual arguments.” OSF Preprints (2021), 10.31219/osf.io/fk3q5.

[28] C. Fong, J. Grimmer, Causal inference with latent treatments. Am. J. Polit. Sci. 10.1111/ajps.12649, (2022).

[29] J. Grimmer, B. M. Stewart, Text as data: The promise and pitfalls of automatic content analysis methods for political texts. Polit. Anal. 21, 267–297 (2013).

[30] M. E. Roberts, B. M. Stewart, D. Tingley, Stm: r package for structural topic models. J. Stat. Softw. 91, 1–40 (2019).

[31] M. Humphreys, R. S. de la Sierra, P. van der Windt, Fishing, commitment, and communication: A proposal for comprehensive nonbinding research registration. Polit. Anal. 21, 1–20 (2013).

[32] M. E. Roberts, B. M. Stewart, D. Tingley, C. Lucas, J. Leder-Luis, S. K. Gadarian, B. Albertson, D. G. Rand, Structural topic models for open-ended survey responses. Am. J. Polit. Sci. 58, 1064–1082 (2014).

[33] S. T. Lanza, D. L. Coffman, S. Xu, Causal inference in latent class analysis. Struct. Equ. Model. 20, 361–383 (2013). 10.1080/10705511.2013.797816 PMC4240500 25419097

[34] A. Volfovsky, E. M. Airoldi, D. B. Rubin, Causal inference for ordinal outcomes. arXiv Preprint arXiv:1501.01234 [stat.ME] (6 January 2015).

[35] J. Lu, P. Ding, T. Dasgupta, Treatment effects on ordinal outcomes: Causal estimands and sharp bounds. J. Educ. Behav. Stat. 43, 540–567 (2018).

[36] R. Pryzant, K. Shen, D. Jurafsky, S. Wagner, Deconfounded Lexicon Induction for Interpretable Social Science, in *Proceedings of the 2018 Conference of the North American Chapter of the Association for Computational Linguistics: Human Language Technologies* (Long Papers, 2018), vol. 1, 1, pp. 1615–1625.

[37] Z. Wood-Doughty, I. Shpitser, M. Dredze, Challenges of Using Text Classifiers for Causal Inference, in *Proceedings of the Conference on Empirical Methods in Natural Language Processing* (Conference on Empirical Methods in Natural Language Processing, 2018:4586. NIH Public Access, 2018). PMC6800252 31633125

[38] A. Feder, N. Oved, U. Shalit, R. Reichart, CausaLM: Causal model explanation through counterfactual language models. Comput. Linguist. 47, 333–386 (2021).

[39] M. L. Anderson, J. Magruder, Split-sample strategies for avoiding false discoveries (National Bureau of Economic Research, 2017).

[40] M. J. van der Laan, S. Rose, *Targeted Learning: Causal Inference for Observational and Experimental Data* (Springer Science & Business Media. 2011).

[41] S. Athey, Machine Learning and Causal Inference for Policy Evaluation, in *Proceedings of the 21th ACM SIGKDD International Conference on Knowledge Discovery and Data Mining* (ACM, 2015), pp. 5–6.

[42] A. Bloniarz, H. Liu, C.-H. Zhang, J. S. Sekhon, B. Yu, Lasso adjustments of treatment effect estimates in randomized experiments. Proc. Natl. Acad. Sci. U.S.A. 113, 7383–7390 (2016). 27382153 10.1073/pnas.1510506113 PMC4941428

[43] W. Zheng, M. J. Van Der Laan, Asymptotic Theory for Cross-Validated Targeted Maximum Likelihood Estimation, in *Targeted Learning: Causal Inference for Observational and Experimental Data*, M. J. van der Laan, S. Rose, Eds. (Springer, 2011).

[44] M. A. Cohen, R. T. Rust, S. Steen, “Measuring Public Perceptions of Appropriate Prison Sentences: Report to National Institute of Justice” (NCJ Report, no. 199365, 2002).

[45] J. Hainmueller, D. J. Hopkins, Public attitudes toward immigration. Annu. Rev. Polit. Sci. 17, 225–249 (2014).

[46] C. Fong, Texteffect: Discovering latent treatments in text corpora and estimating their causal effects (2017).

[47] M. E. Roberts, B. M. Stewart, R. A. Nielsen, Adjusting for confounding with text matching. Am. J. Polit. Sci. 64, 887–903 (2020).

[48] R. Mozer, L. Miratrix, A. R. Kaufman, L. Jason Anastasopoulos, Matching with text data: An experimental evaluation of methods for matching documents and of measuring match quality. Polit. Anal. 28, 445–468 (2020).

[49] K. Keith, D. Jensen, B. O’Connor, Text and causal inference: A review of using text to remove confounding from causal estimates, in *Proceedings of the 58th Annual Meeting of the Association for Computational Linguistics*, (Online: Association for Computational Linguistics, 2020), pp. 5332–5344, 10.18653/v1/2020.acl-main.474.

[50] J. Zhang, S. Mullainathan, C. Danescu-Niculescu-Mizil, Quantifying the causal effects of conversational tendencies, in *Proceedings of the ACM on Human-Computer Interaction 4* (CSCW2, 2020), pp. 1–24.

[51] M. A. Cohen, R. T. Rust, S. Steen, “Measuring Perceptions of Appropriate Prison Sentences in the United States, 2000. ICPSR Version. Nashville, TN: Vanderbilt University [Producer], 2000.” (Ann Arbor, MI: Inter-University Consortium for Political and Social Research.[distributor], 2004).

[52] T. J. Leeper, MTurkR: Access to Amazon Mechanical Turk Requester API via r, 2017.

[53] M. Costa, How responsive are political elites? A meta-analysis of experiments on public officials. J. Exp. Political Sci. 4, 241–254 (2017).

[54] D. M. Blei, Probabilistic topic models. Commun. ACM 55, 77–84 (2012).

[55] J. Hainmueller, D. J. Hopkins, T. Yamamoto, Causal inference in conjoint analysis: Understanding multidimensional choices via stated preference experiments. Polit. Anal. 22, 1–30 (2013).

[56] T. L. Griffiths, Z. Ghahramani, The Indian buffet process: An introduction and review. J. Mach. Learn. Res. 12, 1185–1224 (2011).

[57] F. Doshi, K. Miller, J. V. Gael, Y. W. Teh, Variational inference for the Indian buffet process, in *International Conference on Artificial Intelligence and Statistics* (AISTATS, 2009), pp. 137–144.

