# Supplementary material for: How to make causal inferences using texts
Source: Sci Adv. 2022 Oct 19;8(42):eabg2652. doi: 10.1126/sciadv.abg2652 (PMC9581481; doi:10.1126/sciadv.abg2652)
Supplement: Supplementary file 1 — Supplementary Texts Figs. S1 and S2 Tables S1 to S4 References [file sciadv.abg2652_sm.pdf]

Science Advances
AAAS

Supplementary Materials for

How to make causal inferences using texts

Naoki Egami *et al.*

Corresponding author: Justin Grimmer, jgrimmer@stanford.edu; Margaret E. Roberts, meroberts@ucsd.edu; Brandon M. Stewart, bms4@princeton.edu

Sci. Adv. **8**, eabg2652 (2022)
DOI: 10.1126/sciadv.abg2652

This PDF file includes:

Supplementary Texts
Figs. S1 and S2
Tables S1 to S4
References

Supplementary Text

S1: Selecting *g*

Regardless of the methods used for discovery, the analyst chooses a *g* on the basis of their theoretical question of interest. For example, with candidate biographies, the analyst could use *g* to code whether the candidate is a lawyer or could instead use g to code whether the candidate is a combat veteran. That choice is inherently subjective and depends on which questions the researcher finds most interesting and important. Theoretical interest is the first and most fundamental desirable property of *g*.

Property 1: Theoretical Interest

g should generate low-dimensional representations of text that operationalize concepts from a theory so that researchers can test the theory's observable implications. Ideally, we would like to focus on causal effects with large magnitude, because larger effects help us to explain more of the behavior of theoretical interest.

However, once we have defined a question of theoretical interest, there are three further properties that make one g more attractive than others interpretability, label fidelity, and tractability.

Property 2: Interpretability

The meaning of the codebook's labels or scores should be communicable by human beings. If *g* assigns binary labels, those labels are interpretable if a human being can provide an explanation for what the two categories mean. If *g* assigns continuous scores, the researcher should be able to communicate what a score of 1.4 rather than -0.3 means.

Property 3: Label Fidelity

The low-dimensional representations of text should accurately capture the property implied by the label. This is a common exercise in the social sciences; there is always an implicit mapping between the labels we use for our variables and the reality of what our labels measure. For text analysis, we think of maximizing label fidelity as minimizing the surprise that a reader would have in going from the label to reading the text. Fidelity is closely connected to the literature on validity in measurement and manual content analysis (see e.g., *15, 29, 22*).

Property 4: Tractability

Finally, we want the development and deployment of *g* to be tractable. In the context of manual content analysis this means the codebook can be applied accurately and reliably by human coders and that the number of documents to be coded is feasible for the resources available. In the case of learning g statistically, tractability implies that we have a model which can be estimated using reasonable computational resources and that it is able to learn a useful representation with the number of documents we possess.

In short, theoretical interest measures the closeness between the label and the theoretical context, interpretability measures the closeness between the label and the reader's understanding, fidelity measures the closeness between the label and the raw text, and tractability measures how practical the procedure is.

There is an inherent tension between the four properties. This is most acute with the tension between theoretical interest and label fidelity. It is often tempting to assign a very general label even though g in fact measures a more specific concept within the text. This increases theoretical relevance, but lowers fidelity. The consequence can be research that is more difficult to replicate. Alternatively, we might have a g that coincides with a label because it increases the chances that our result can be replicated. But this could reduce the theoretical interest.

The challenges of measurement and representation are not new — the analog of g lurks in every research design, including those that use standard data. When making an argument, the researcher needs to find empirical surrogates or operationalize the concepts in her theoretical argument. For example, when a researcher uses change in the consumer price index, she is projecting a high-dimensional and complicated phenomenon – inflation – into a lower-dimensional and more tractable index. The causal estimand is defined in terms of its effect on the index, but the theoretical argument is about inflation. This tension exists in reductions of all manner of complex political and social phenomena from the economy to forms of governance to political attitudes. While there is no single correct choice in each case, the reader can and should still interrogate the degree to which the chosen measure appropriately captures the researcher's broader theoretical concept.

S2: Proofs and Technical Details

It might seem natural to inquire about the properties of the estimator we use to obtain g. In this setting, we can use the procedure to obtain g as an estimator G. If we suppose that there is some true function $\breve{g}$ we might ask how well our estimator G performs—in large samples does the g converge to $\breve{g}$ and in small samples how discrepant is g compared to $\breve{g}$?

While it is certainly useful to conceive of the estimator G, it is misguided to suppose that there is a true $\breve{g}$ for some data set that our procedure is attempting to reveal. To see why it is not useful to suppose there is a true function $\breve{g}$ consider a hypothetical experiment where we examine how people respond to a knock on the door and encouragement to vote. We might be immediately interested in whether respondents are more likely to express a positive tone about political participation. To investigate this, we might construct a g that measures the tone of open-ended responses. But, we might also be interested in the topics that are discussed after receiving a mobilization, or whether individuals mention privacy concerns. There is also large variation in the ways we might examine how the particular contents of the mobilization message might affect respondents. We might be interested in whether messages that have a positive tone are more likely to increase turnout, whether highlighting the threats from a different political party causes an increase in turnout, or whether threatening the revelation of voter history to neighbors is the most effective method of increasing turnout. This hypothetical example makes clear that there is no “true" application-independent function for obtaining either the dependent variable or treatment when making causal inferences from texts. Further, the fact that we need to discover g at all implies that as the researcher we might be unsure about what properties we want g to have—making it particularly difficult to evaluate the estimator *a priori*.

Proof: Identifying ATE with text as dependent variable

This appendix section proves that after using the codebook function g on text as a dependent variable the ATE is still preserved. We then weaken conditions needed on g to identify the ATE.

We make Assumption 1-3 and we suppose that we have a codebook function g. Without loss of generality we will suppose that the codebook function maps text into a set of K categories with the constraint that the sum across all categories is equal to 1. One example of this is using an STM to estimate the dependent variables from a set of texts. Suppose further that we are interested in the effect of a dichotomous intervention on the prevalence of the kth category. Our formal estimand of interest, then, is:

$$\text{ATE}_k \quad = \quad \text{E}[z_{i,1,k} - z_{i,0,k}].$$

Where $z_{i,1,k}$ corresponds to the prevalence of the kth category for observation i after receiving $T_i = 1$.

We can see that the treatment effect is still identified by noting that after our randomization we have

$$\begin{aligned} & \mathrm{E}\big[g\big(\mathbf{Y}_i(T_i = 1)\big)|T_i = 1\big] - \mathrm{E}\big[g\big(\mathbf{Y}_i(T_i = 0)\big)|T_i = 0\big] \\ = & \quad \mathrm{E}\big[z_{i,1,k}|T_i = 1\big] - \mathrm{E}\big[z_{i,0,k}|T_i = 0\big] \\ = & \quad \mathrm{E}\big[z_{i,1,k} - z_{i,0,k}\big] = \mathrm{ATE}_k \end{aligned}$$

Where we apply the definition of g and the randomization of the treatments. Note that for this proof to work, it is essential that g is fixed, otherwise the expectation is undefined.

We can make a slightly weaker requirement of g and still preserve identification of the causal effect. Specifically, the only requirement is that any potential other g, $\tilde{g}$ agrees with g for category k for all text documents, or that $\tilde{g}(\mathbf{Y})_k = g(\mathbf{Y})_k$ for all $\mathbf{Y} \in \mathcal{Y}$. This implies the other categories could be arbitrarily different, but logically it requires that the total proportion of documents placed in the other $K - 1$ categories is equal for both functions. The proof follows immediately from the (obvious) proof above.

Technical Definition of the Fundamental Problem of Causal Inference with Latent Variables

In this section we offer a formal definition of the Fundamental Problem of Causal Inference with Latent Variables. To formally define the FPCILV we rewrite g as explicitly dependent on training data: both treatments $\mathbf{T_J}$ and responses $\mathbf{Y_J}$. Specifically, we will write the value of $g_\mathbf{J}$ for observation i that received treatment $\mathbf{T}_i$ as $g\left(\mathbf{Y}_i(\mathbf{T}_i); \mathbf{Y_J}(\mathbf{T_J})\right)$ where $\mathbf{Y_J}(\mathbf{T_J})$ describes all respondents' text-based responses and the vector of treatments for everyone in the set $\mathbf{J}$. Suppose now that we re-randomize treatment $\mathbf{T}'_\mathbf{J}$, such that $\mathbf{T}_i = \mathbf{T}'_i$ and that $\mathbf{T}_j \neq \mathbf{T}'_j$ for at least one $j \in \mathbf{J} \backslash i$. Further, suppose we obtain new responses $\mathbf{Y_J}(\mathbf{T}'_\mathbf{J})$.

The FPCILV emerges if $g_\mathbf{J}(\mathbf{Y}_i(\mathbf{T}_i)) \equiv g\left(\mathbf{Y}_i(\mathbf{T}_i); \mathbf{Y_J}(\mathbf{T_J})\right) \neq g\left(\mathbf{Y}_i(\mathbf{T}'_i); \mathbf{Y_J}(\mathbf{T}'_\mathbf{J})\right) \equiv g_\mathbf{J}(\mathbf{Y}_i(\mathbf{T}'_i))$ even though $\mathbf{Y}_i(\mathbf{T}_i) = \mathbf{Y}_i(\mathbf{T}'_i)$. In plain language, the lower dimensional representation of document i is different between the two randomizations even though the texts themselves are the same. This is particularly problematic if we wanted to characterize the bias in estimators, or their properties in large samples. This is because expectations are taken over different treatment allocations. And different treatment allocations, under many different procedures for obtaining a codebook function g, imply that there are new categories of the dependent variable or new treatments in the text.

Sufficient Assumptions to Resolve the FPCILV

Formally, to assume away the FPCILV we would assume that $g_\mathbf{J}\left(\mathbf{Y}_i(\mathbf{T}_i); \mathbf{Y_J}(\mathbf{T_J})\right) = g_\mathbf{J}\left(\mathbf{Y}_i(\mathbf{T}'_i); \mathbf{Y_J}(\mathbf{T}'_\mathbf{J})\right)$ for all $\mathbf{T_J}, \mathbf{T}'_\mathbf{J}$ and all $i \in \mathbf{J}$. For the text-as-outcome case, if we assume that the treatment has no effect on text-based outcomes, $\mathbf{Y_J}(\mathbf{T_J}) = \mathbf{Y_J}(\mathbf{T}'_\mathbf{J})$, which implies the absence of the FPCILV. However, the general conditions for this stability can be difficult to obtain (*21*). This assumption also does not solve the problem of overfitting.

S3: Further Connections to Literature

In this section we provide a further connection to the machine learning literature. To make the connection, we compare our sequential approach to other methods for ensuring that we avoid overfitting. One natural approach would be to adopt a cross-fitting or cross-validation approach which has been extremely successful in other contexts (*21, 38*). In k-fold cross validation the data is partitioned into k equally sized partitions. The model is trained on all but one of these partitions (called the held-out set) and then model is estimated on the held-out set. Then the procedure is repeated so each of the k partitions is treated as the held-out set at least once. This forms an estimate for every observation i where the prediction comes from a model which was not trained on observation i. This is a powerful approach but relies on the idea that the predictions will be comparable across observations which is true, for example, in settings where the estimand is well-defined in advance of the split. In our setting, though, we have two problems that preclude the use of cross validation. First, when a human is in the loop there is no way to separate the model fitting procedures because the human will remember the insights from the previous train-test split. Second, because the estimand is not defined in advance of the split, every fold of the cross-validation could result in us measuring slightly different concepts. Thus, we would have no coherent way to align the g across the cross validation folds. Taken together, this suggests that a cross-validation or cross-fitting strategy could only be pursued under strong assumptions about the existence of a true g or with severe limitations on the discovery process.

S4: Explanation of Procedure

The following steps are a road map for our procedure.

1. **Collect** a set of documents and split them into a training set and a test set. Do not look at the test set.
2. Using your training set only, **choose** g that compresses the high-dimensional text to a low-dimensional variable that will serve as either your treatment or outcome. Assign labels to low-dimensional categories.
3. **Validate** that the chosen g accurately maps to a concept of theoretical significance for your argument.
4. **Estimate** the causal effect using the test set with the g discovered in test set. You can only use the test set once.
5. **Validate** that the g worked as expected in the test set.
6. Ideally, **replicate** your findings in a new sample, repeating steps 1-5. If you are unable to replicate, clarify what you would alter in the next experiment.

S5: Uncertainty Estimation with g

Once we have applied g to our test data we can calculate confidence intervals using usual variance estimators that capture uncertainty about our estimate given a limited sample size conditional on g. Examples in prior work tends to explicitly take the view of $g(\mathbf{Y})$ as a latent variable about which there is some additional measurement uncertainty and advocated approaches to incorporate this additional uncertainty into our confidence intervals (*17,43*). For example, Roberts *et. al.* (*43*) advocates a simulation approach to integrate over the variational approximation to the posterior distribution which conditions on the learned topic-word distribution, but accounts for the fact that the document-topic proportion $\boldsymbol{\theta}$ cannot be known with certainty for a particular document because it has a finite length. Fong and Grimmer (*17*) use a bootstrap approach which captures measurement uncertainty both in the topic-word parameters and the document-topic representation. While this approach is intuitively appealing, it complicates the definition of g as a function because we run the risk of the same text mapping to two different values of the latent variable (failing the vertical line test). In the interest of simplicity we do not include this form of measurement error in this article and leave to future work the incorporation of this uncertainty into the causal framework

S6: Structural Topic Model

The Structural Topic Model is a mixed membership model of texts related to Latent Dirichlet Allocation (*54*) which was developed in Roberts *et. al.* (*43*) and implemented in the stm package in R (*30*). It allows for the analyst to incorporate observed document metadata which is able to affect either topical prevalence (the amount which a topic is discussed) and topical content (the way in which a topic is discussed). In this paper we consider the case in which a set of observed metadata which includes the treatment and pre-treatment covariates are allowed to affect topic prevalence and there are no topical content covariates. Denoting the pretreatment covariates for document i as $\mathbf{X}_i$ and the scalar treatment as T_i, the generative process can be given as:

$$\begin{aligned} \boldsymbol{\eta}_i &\sim \text{Normal}(\mathbf{X}_i \boldsymbol{\gamma}_X + T_i \gamma_\tau, \boldsymbol{\Sigma}) \\ \theta_{i,k} &= \frac{\exp(\eta_{i,k})}{\sum_{k=1}^{K} \exp(\eta_{d,k})} \\ z_{i,n} &\sim \text{Categorical}(\boldsymbol{\theta}_i) \\ w_{i,n} &\sim \text{Categorical}(\boldsymbol{\beta}_{z_{i,n}}) \end{aligned}$$

Where $\boldsymbol{\theta}_d$ is a K-dimensional vector on the simplex indicating the proportion of the document allocated to each topic formed by applying the softmax function to $\boldsymbol{\eta}_d$ a vector in $\mathcal{R}^{K-1}$ where the K-th element is fixed to zero. $z_{i,n}$ is a token level latent variable containing the assignment for token n of document i. $\boldsymbol{\beta}$ is a K by V dimensional matrix where each row contains the conditional probability of seeing word v given that is about topic k. The model differs from Latent Dirichlet Allocation in its use of a logistic normal prior distribution for the document-topic proportions and through the ability to have that prior centered at a document-specific location determined by the document metadata.

The model is estimated using partially-collapsed, non-conjugate, variational inference. $\boldsymbol{\gamma}$ and $\boldsymbol{\Sigma}$ are given regularizing priors of the user's choice and β is point estimated. The model optimization problem is non-convex and so a careful initialization strategy is necessary.

Obtaining and using g

In a given experiment we employ the following steps:

1. Create the train-test split
2. In the training set (discovery)
   - explore the documents as desired using STM
   - choose an estimand (including assigning and validating a label)
   - Identify the mapping function g such that

$$\widehat{\boldsymbol{\theta}}_\iota = g(\mathbf{Y}_i, \widehat{\boldsymbol{\beta}}, \widehat{\boldsymbol{\mu}}_\iota, \widehat{\boldsymbol{\Sigma}})$$

3. In the test set (evaluation)
   - Using g, obtain our transformed outcome for each document. (see below for details)
   - Estimate treatment effects (using for example the difference of means)
   - Validate model fit and label fidelity in the test set.

Application of g in STM is equivalent to predicting $\boldsymbol{\theta}_\mathbf{i}$ for a held-out document i. This can be accomplished with the recently added fitNewDocuments function in the stm package. In the STM model, the latent variable $\boldsymbol{\theta}_\mathbf{i}$ is a function of a global prior $(\boldsymbol{\mu}, \boldsymbol{\Sigma})$, the topic word parameters $\boldsymbol{\beta}$ and the observed words $\mathbf{W_d}$. The token-level latent variables $\mathbf{Z}$ are integrated out. We have estimated $\boldsymbol{\beta}$ in the train set and in many ways this communicates what the topics substantively contain. We must also decide how to set our priors $\boldsymbol{\mu}$ and $\boldsymbol{\Sigma}$.

The stm package offers three options: no prior, the covariate-specific prior and the average prior. The 'no prior' setting sets $\boldsymbol{\mu}$ to a vector of zeroes and $\boldsymbol{\Sigma}$ to be a diagonal matrix with very large diagonals. The covariate-specific prior uses the observed covariates in the new documents to construct the document-specific prior. The average prior averages over the values of $\boldsymbol{\mu}$ in the training set and provides a single average prior for all documents. Formally, we take the column means of the D by $K-1$ matrix $\boldsymbol{\mu}$ in the training set which we call $\widetilde{\boldsymbol{\mu}}$. We then recalculate $\boldsymbol{\Sigma}$ as though the update had been made using the new value of μ. The update is then $\widetilde{\boldsymbol{\Sigma}} = \boldsymbol{\Sigma} - (\sum_d (\boldsymbol{\eta}_\mathbf{d} - \boldsymbol{\mu}_\mathbf{d})\,(\boldsymbol{\eta}_\mathbf{d} - \boldsymbol{\mu}_\mathbf{d})^T) + (\sum_d (\boldsymbol{\eta}_\mathbf{d} - \widetilde{\boldsymbol{\mu}}_d)\,(\boldsymbol{\eta}_\mathbf{d} - \widetilde{\boldsymbol{\mu}}_d)^T)$.

If we have used only pre-treatment covariates in the STM model we can use any of these strategies. In our application we do include the treatment and so we cannot use the covariate-specific prior because then the same text would yield two different values of the outcome depending on the treatment assignment. For our application we use the average prior. When using a version of g which is not the covariate-specific prior, we recommend that analysts assess effects in the training set using the same procedure as in the test set. While the effects will generally not be very different (particularly for long documents), maintaining the same procedure should provide a better expectation of test set behavior. For example, in our application Figure S1 compares our training set estimates using both the covariate-specific prior and the averaged prior and compares them to the test set (which uses the averaged prior). Using the average prior to make predictions in the training set before calculating effect estimates gives us a better indication of what we will eventually observe in the test set.

S7: Supervised Indian Buffet Process

Additional estimands

The analyst might also be interested in estimating the effect of an interaction between two components k and l. For example, the researcher might be interested if including military service into a candidate profile has a different effect on candidate ratings if the profile also includes that the candidate is female. This could be estimated as the Average Component Interaction Effect (ACIE) (*55*):

$$\begin{aligned} ACIE_{k,l} \quad &= \quad \int_{Z_{-k,-l}} E\,[\left(Y\left(Z_k = 1, Z_l = 1, Z_{-k,-l}\right) - Y\left(Z_k = 1, Z_l = 0, Z_{-k,-l}\right)\right) \\ &- \quad \left(Y\left(Z_k = 0, Z_l = 1, Z_{-k,-l}\right) - Y\left(Z_k = 0, Z_l = 0, Z_{-k,-l}\right)\right] m\left(Z_{-k,-m}\right) dZ_{-k,-m} \end{aligned}$$

The ACIE will be the difference between the AMCE for military service for a candidate description that includes information that the candidate is female and the AMCE for military service for a candidate description that does not include this information.

Note that the three complications from the last section also pertain to the case of multidimensional treatments. If the mapping g between **T** and **Z** is not known before defining and reading the treatment texts or the outcome is used in the estimation of these mapping, then the FPCILV is present. Even when using hand coding, researchers should either use a pre-test to determine their coding scheme or use a training/test split to first learn a coding scheme using the responses and then separately estimate the treatment effects.

The argument for binary features

In this section we explain why we use binary features of texts in order to estimate causal effects. A different approach to estimating the function g would be to estimate real valued features that explain the text well, such as the principal components of a document term matrix or some other low-dimensional embedding of the observations. Using these real valued embeddings for **Z**, the impact of **Z** on **Y** can be estimated directly. Using real valued features of documents, however, causes several problems that leads us to use binary features instead. First, many methods for discovering real valued features incorporate information about the text, but not the response. For example, we might use the loadings on principal components to describe text-treatments. This can lead to the discovery of features that explain the content of texts but *do not* explain the response to those texts and therefore are not particularly useful for causal inference. This makes clear that our goal should be to find a low-dimensional representation that explains both the texts and the response well. Second, using real valued features requires the imposition of a stringent set of functional form assumptions. This is because even flexibly estimating the response to some continuous feature requires some guidance from a model. And the more flexible the fit, the more data needed to credibly estimate the response to the continuous treatment. And as the number of included factors increases, the curse of dimensionality makes it all but impossible to fit anything other than a linear regression. Alternative approaches, such as an Indian Buffet Process (*56*), yield a binary feature vector about the treatments that are present or absent in a text, but fail to include information about the responses.

Given the issue with continuous treatments and the importance of including information about the response, we use a method that finds latent features and observation's binary loading on those features, which are then used to estimate treatment effects. Fong and Grimmer (*17*) create an unsupervised method for estimating treatments from text data and the responses. They develop a supervised Indian Buffet Process (sIBP) that discovers the topics within the documents that are related to the outcome. The authors assume that the proportion of documents in each latent feature k is π_k, where π_k is generated by a stick-breaking algorithm (*57*). Each document can be summarized by treatment vector Z_j where $z_{j,k} \sim \text{Bernoulli}(\pi_k)$. Note that because each individual $z_{j,k}$ is drawn from a Bernoulli that a treatment document can have more than one latent feature, allowing for multi-dimensional treatments.

The authors assume a mapping from Z_i to the standardized term-document matrix X_i through the D-dimensional vector A_k, where $X_i \sim \text{MvtNormal}(Z_i A, \sigma_n^2 I_D)$. The latent feature vector Z_i also affects the response Y_i through the normal, $Y_i \sim \text{Normal}(Z_i \beta, \tau^- 1)$ where $\tau \sim \text{Gamma}(a, b)$. Thus with the model the authors both want to discover the latent treatments Z_i *and* estimate their influence on the outcome by estimating β. The authors use variational approximation to estimate these parameters.

Fong and Grimmer (*17*) apply the sIBP to the training data in order to learn g. In the test set, Fong and Grimmer (*17*) use g to infer the treatments that are present in a particular text, but alter the inference to avoid conditioning on the dependent variable. They do this because otherwise the inferred treatments present in the test set will depend upon the observation's response to that text, which creates obvious problems for causal inference.

Once the latent treatments are inferred in the test set documents, their effect can be estimated using any procedure that might be used to analyze an experiment. Fong and Grimmer (*17*) use a simple linear regression with each of the latent features as the regressors to estimate the effects of the treatments. More complicated models could be used to estimate interactions or to extrapolate effects to a different population of documents.

S8: Immigration Experiments

Additional details of the immigration experiments are reported in Figures S1-S2 and Tables S1-S4.

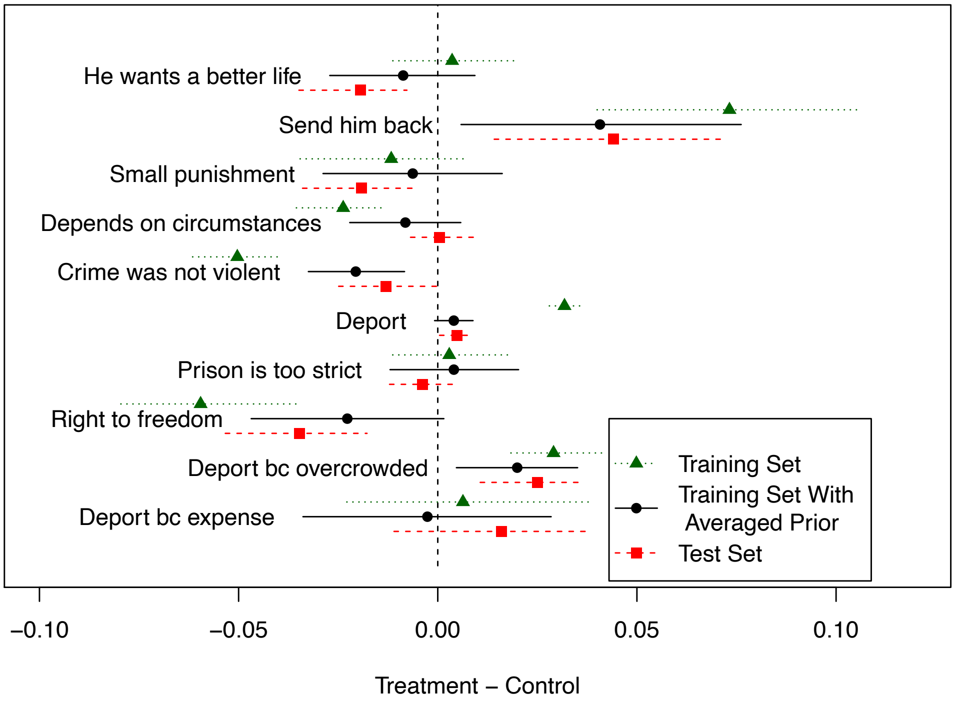

Fig. S1.

Train-Test set effect in Experiment 1 comparing g using the model estimates (training set), the training set with averaged prior and the test set. Note that while the estimates are broadly similar, in general the training set with averaged prior is a closer approximation to what we end up seeing.

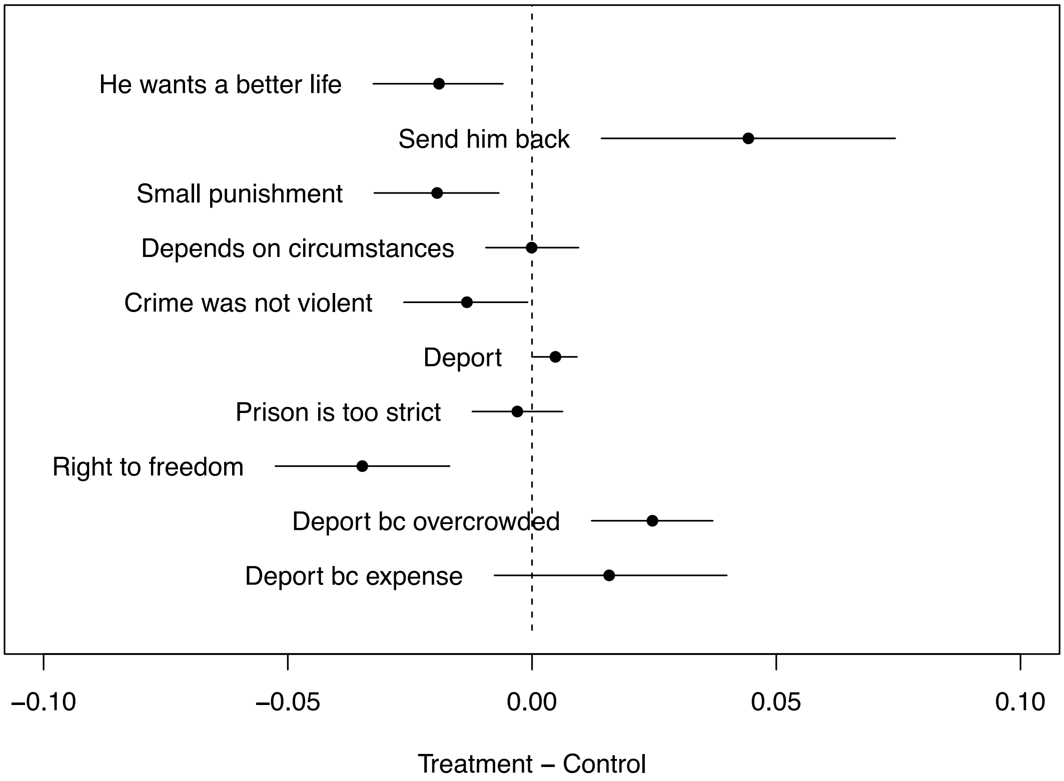

Fig. S2.

Test Set results for Experiment 1. Point estimates and 95% *confidence intervals.*

Table S1.
Data Source, Dates, and Number of Participants for Three Immigration Experiments.

Exp. #	Data Source	Date	N
1	Cohen *et. al.* (*51)*	May 16-August 8, 2000	1,300
2	Mechanical Turk	June 30-July 16, 2017	1,299
3	Mechanical Turk	September 10, 2017	1,094

Table S2.

Experiment 1, Words most representative of topics.

	Label	Highest Probability Words
Topic 1	He wants a better life	didnt, want, pay, better, life, probabl, isnt
Topic 2	Send him back	back, countri, send, home, well, charg, troubl
Topic 3	Small punishment	offens, reason, like, chanc, first, can, citizen
Topic 4	Depends on circumstances	come, depend, doesnt, free, feel, law, shouldnt
Topic 5	Crime was not violent	crime, commit, violent, immigr, wasnt, look, never
Topic 6	Deport	deport, that, give, counti, peopl, look, guilti
Topic 7	Prison is too strict	enter, anyth, right, live, realli, illeg, anybodi
Topic 8	Right to freedom	just, tri, get, hes, came, freedom, put
Topic 9	Deport bc overcrowded	sent, prison, think, alreadi, anoth, done, hasnt
Topic 10	Deport bc expense	dont, think, know, time, need, serv, crimin

Table S3.

Experiment 1: selected representative documents of each topic.

	Label	Representative Document
Topic 1	He wants a better life	we’re the land of opportunity everybody wants a better life
Topic 2	Send him back	send him back to his country
Topic 3	Small punishment	"it was his first offense, didn’t hurt anybody, maybe a fine though, probation or something. that’s nice serious like murder or robbery"
Topic 4	Depends on circumstances	it depends on reaason why he is coming into state if he was coming to beter himself its ok if he has a record he should be disbarred or deported
Topic 5	Crime was not violent	because he didnt commit a crime that was effecting someone else’s individual liberties
Topic 6	Deport	he should be deported
Topic 7	Prison is too strict	because he didnt do anything except illegally enter
Topic 8	Right to freedom	Because he’s just trying to get his freedom. Maybe he’s trying to away from a tough situation/that country-maybe it’s not good for him.
Topic 9	Deport bc overcrowded	he should be sent to prison in another country our prisons are over crowded already
Topic 10	Deport bc expense	because i think he shold be deported-p-i don’t think he should be supported in our prison system and i don’t think he should be allowed to immigrate here

Table S4.

Experiment 3: Topics and representative documents

	Label	Representative Document
Topic 1	Limited punishment with help to stay in country, complaints about immigration system	with all of the ""exceptional america"", ""anyone can get rich"" propaganda this country throws out(not exactly the truth since we are no longer exceptional(literacy, happiness, health care), and the fact some people are actually taking us backwards........who can blame these people for trying? And, if we are talking about people from south america, it is our interference and OUR drug war that is making the area dangerous and poor and people dont want to live there! We shpould welcome them with open arms since we made a mess of their country!! I dont think we should do anything to some of these people. Especially if they have been here for awhile, certainly not prison!!!!
Topic 2	Deport	I think they will probably be detained long enough awaiting trail and deportation and shouldn't serve any extra incarceration. I do not believe that process of trial and deportation would be instantaneous and I do not think that there needs to be and deterrent of extra jail time awarded if they are already going through the trial of being deported back to their home country.
Topic 3	Deport because of money	I am favor of just sending him back. Enough wasting tax payers money. Him living in USA prison is actually a higher standard of living than his country. He gets room and food everyday.
Topic 4	Depends on the circumstances	My first answer is no, but it also depends on why he illegally entered the U.S. If he committed a crime and fled to the U.S. then yes he should. If he came here for a better life, then I think that is something to be commended rather than punished. The people who would go that far to get better in life show hard work and dedication which America is supposed to be founded on. If I was a business owner, that is a man I would hire because he would strive for the best to keep his job because it meant a better life for him.
Topic 5	More information needed	She did commit a crime but there could be a legitimate reason as to why she did so. She could be held until her background is checked and carefully monitored as to where bouts and work for so long and required to become a gainful citizen as everyone else.

	Label	Representative Document
Topic 6	Crime, small amount of jail time, then deportation	It doesn’t seem as though the man poses a threat, so I’m reluctant to say that he deserves to be imprisoned. He did, however, enter the country illegally. When actual citizens break the law, they are sentenced to jail time, so I don’t see why it should be any different with others. Also, if I were caught entering another country illegally, I would fully expect to face serious legal consequences.
Topic 7	Punish to full extent of the law	This person broke a law so that means they should be punished accordingly. Despite this person’s history, this individual did something illegal and as with anyone else, they must serve the applicable sentence for the crime.
Topic 8	Allow to stay, no prison, rehabilitate, probably another explanation	We do not know what is her real situation. I have a friend graduated from one of the Ivy league schools, she taught in one universities in USA, her visa was expired just because she waited adjustment from Immigration, that means was not her fault at all, but at the end court called her, she had to be in court for several times before she decided to go home to her native country. Base on what she said, Immigration made tough access for skilled and educated people, they prefer illegal people with children. Therefore, government need to do something to fix this corrupt system.
Topic 9	No prison, deportation	he should be deported once again instead of being kept in prison and using our resources, it does not seem that he will be productive after another prison sentence
Topic 10	Should be sent back	I feel this person should be sent back to his own country. I do not know of any punishment that would improve the situation. If we imprison him in this country, we would have to accommodate him and pay for his food and essentials. I feel that would cost far more than the cost of deporting him back to his country.
Topic 11	Repeat offender, danger to society	This man appears to be disturbed in that he enters this country illegally and commits crimes while here. I believe this person has a distorted view of how to live in this world and I do not think that he wants help nor does he want to live a law abiding life in the U.S. He also, appears to be an obvious threat to others. Prison will probably not discourage this individual from entering illegally but a prison sentence might send a stronger message than simply being deported. He did violate our laws when entering the country without permission. This person’s home country

Label	Representative Document
	should step up and begin taking responsibility for their citizens and should try to monitor individuals deported back to the home country.

REFERENCES AND NOTES

1. N. Gandhi, W. Zou, C. Meyer, S. Bhatia, L. Walasek, Computational methods for predicting and understanding food judgment. *Psychol. Sci.* **33**, 579–94 (2022).

2. S. Bhatia, C. Y. Olivola, N. Bhatia, A. Ameen, Predicting leadership perception with large-scale natural language data." *Leadersh. Q.* 101535 (2021).

3. M. Bertrand, D. Karlan, S. Mullainathan, E. Shafir, J. Zinman, What's advertising content worth? Evidence from a consumer credit marketing field experiment. *Q. J. Econ.* **125**, 263–305 (2010).

4. J. Berger, A. Humphreys, S. Ludwig, W. W. Moe, O. Netzer, D. A. Schweidel, Uniting the tribes: Using text for marketing insight. *J. Mark.* **84**, 1–25 (2020).

5. Myers, Kyle. 2020. The elasticity of science. *Am. Econ. J. Appl. Econ.* **12** (4): 103–34.

6. S. Bhatia, R. Richie, Transformer networks of human conceptual knowledge. PsyArXiv. 13 November 2020.

7. A. E. Boydstun, *Making the News: Politics, the Media, and Agenda Setting* (University of Chicago Press, 2013).

8. A. Catalinac, From pork to policy: The rise of programmatic campaigning in Japanese elections. *J. Polit.* **78**, 1–18 (2016).

9. A. Spirling, U.S. treaty making with American Indians: Institutional change and relative power, 1784–1911." *Am. J. Polit. Sci.* **56**, 84–97 (2012).

10. D. B. Rubin, Estimating causal effects of treatments in randomized and nonrandomized studies. *J. Educ. Psychol.* **66**, 688–701 (1974).

11. J. Pearl, *Causality* (Cambridge Univ. Press, 2009).

12. I. Lundberg, R. Johnson, B. M. Stewart, What is your estimand? Defining the target quantity connects statistical evidence to theory *Am. Sociol. Rev.* **86**, 532–565 (2021).

13. M. Laver, K. Benoit, J. Garry, Extracting policy positions from political texts using words as data. *Am. Polit. Sci. Rev.* **97**, 311–331 (2003).

14. J. W. Pennebaker, M. R. Mehl, K. G. Niederhoffer, Psychological aspects of natural language use: Our words, our selves. *Annu. Rev. Psychol.* **54**, 547–577 (2003).

15. K. M. Quinn, B. L. Monroe, M. Colaresi, M. H. Crespin, D. R. Radev, How to analyze political attention with minimal assumptions and costs. *Am. J. Polit. Sci.* **54**, 209–228 (2010).

16. G. W. Imbens, D. B. Rubin. *Causal Inference in Statistics, Social, and Biomedical Sciences* (Cambridge Univ. Press, 2015).

17. C. Fong, J. Grimmer, Discovery of Treatments from Text Corpora, in *Proceedings of the 54th Annual Meeting of the Association for Computational Linguistics* (Long Papers, 2016), vol. 1, pp. 1600–1609.

18. J. Robins, L. Li, E. Tchetgen, A. van der Vaart, Higher order influence functions and minimax estimation of nonlinear functionals, in *Probability and Statistics: Essays in Honor of David A. Freedman* (Institute of Mathematical Statistics, 2008), pp. 335–421.

19. S. Wager, S. Athey, Estimation and inference of heterogeneous treatment effects using random forests. *J. Am. Stat. Assoc.* **113**, 1228–1242 (2018).

20. M. Fafchamps, J. Labonne, Using split samples to improve inference on causal effects. *Polit. Anal.* **25**, 465–482 (2017).

21. V. Chernozhukov, D. Chetverikov, M. Demirer, E. Duflo, C. Hansen, W. Newey, J. Robins, Double/debiased machine learning for treatment and structural parameters. *J. Econom.* **21**, C1–C68 (2018).

22. K. Krippendorff, *Content Analysis: An Introduction to Its Methodology* (Sage, 2004).

23. D. B. Rubin, Comment on "randomization analysis of experimental data: The fisher randomization test" by D. Basu. *J. Am. Stat. Assoc.* **75**, 591–593 (1980).

24. A. E. Hubbard, S. Kherad-Pajouh, M. J. van der Laan, Statistical inference for data adaptive target parameters. *Int. J. Biostat.* **12**, 3–19 (2016).

25. J. P. Simmons, L. D. Nelson, U. Simonsohn, False-positive psychology: Undisclosed flexibility in data collection and analysis allows presenting anything as significant. *Psychol. Sci.* **22**, 1359–1366 (2011).

26. L. Vavreck, *The Message Matters* (Princeton Univ. Press, 2009).

27. J. G. Voelkel, M. Malik, C. Redekopp, R. Willer, "Changing Americans' attitudes about immigration: Using moral framing to bolster factual arguments." OSF Preprints (2021), https://doi.org/10.31219/osf.io/fk3q5.

28. C. Fong J. Grimmer, Causal inference with latent treatments. *Am. J. Polit. Sci.* 10.1111/ajps.12649 (2022).

29. J. Grimmer, B. M. Stewart, Text as data: The promise and pitfalls of automatic content analysis methods for political texts. *Polit. Anal.* **21**, 267–297 (2013).

30. M. E. Roberts, B. M. Stewart, D. Tingley Stm: R package for structural topic models. *J. Stat. Softw.* **91**, 1–40 (2019).

31. M. Humphreys, R. S. de la Sierra, P. van der Windt, Fishing, commitment, and communication: A proposal for comprehensive nonbinding research registration. *Polit. Anal.* **21**, 1–20 (2013).

32. M. E. Roberts, B. M. Stewart, D. Tingley, C. Lucas, J. Leder-Luis, S. K. Gadarian, B. Albertson, D. G. Rand, Structural topic models for open-ended survey responses. *Am. J. Polit. Sci.* **58**, 1064–1082 (2014).

33. S. T. Lanza, D. L. Coffman, S. Xu, Causal inference in latent class analysis. *Struct. Equ. Model.* **20**, 361–383 (2013).

34. A. Volfovsky, E. M. Airoldi, D. B. Rubin, Causal inference for ordinal outcomes. arXiv Preprint arXiv:1501.01234 [stat.ME] (6 January 2015).

35. J. Lu, P. Ding, T. Dasgupta, Treatment effects on ordinal outcomes: Causal estimands and sharp bounds. *J. Educ. Behav. Stat.* **43**, 540–567 (2018).

36. R. Pryzant, K. Shen, D. Jurafsky, S. Wagner, Deconfounded Lexicon Induction for Interpretable Social Science, in *Proceedings of the 2018 Conference of the North American Chapter of the Association for Computational Linguistics: Human Language Technologies* (Long Papers, 2018), vol. 1, 1, pp. 1615–1625.

37. Z. Wood-Doughty, I. Shpitser, M. Dredze, Challenges of Using Text Classifiers for Causal Inference, in *Proceedings of the Conference on Empirical Methods in Natural Language Processing* (Conference on Empirical Methods in Natural Language Processing, 2018:4586. NIH Public Access, 2018).

38. A. Feder, N. Oved, U. Shalit, R. Reichart, CausaLM: Causal model explanation through counterfactual language models. *Comput. Linguist.* **47**, 333–386 (2021).

39. M. L. Anderson, J. Magruder, Split-sample strategies for avoiding false discoveries (National Bureau of Economic Research, 2017).

40. M. J. van der Laan, S. Rose, *Targeted Learning: Causal Inference for Observational and Experimental Data* (Springer Science & Business Media. 2011).

41. S. Athey, Machine Learning and Causal Inference for Policy Evaluation, in *Proceedings of the 21th ACM SIGKDD International Conference on Knowledge Discovery and Data Mining* (ACM, 2015), pp. 5–6.

42. A. Bloniarz, H. Liu, C.-H. Zhang, J. S. Sekhon, B. Yu, Lasso adjustments of treatment effect estimates in randomized experiments. *Proc. Natl. Acad. Sci. U.S.A.* **113**, 7383–7390 (2016).

43. W. Zheng, M. J. Van Der Laan, Asymptotic Theory for Cross-Validated Targeted Maximum Likelihood Estimation, in *Targeted Learning: Causal Inference for Observational and Experimental Data*, M. J. van der Laan, S. Rose, Eds. (Springer, 2011).

44. M. A. Cohen, R. T. Rust, S. Steen, "Measuring Public Perceptions of Appropriate Prison Sentences: Report to National Institute of Justice" (NCJ Report, no. 199365, 2002).

45. J. Hainmueller, D. J. Hopkins, Public attitudes toward immigration. *Annu. Rev. Polit. Sci.* **17**, 225–249 (2014).

46. C. Fong, Texteffect: Discovering latent treatments in text corpora and estimating their causal effects (2017).

47. M. E. Roberts, B. M. Stewart, R. A. Nielsen, Adjusting for confounding with text matching. *Am. J. Polit. Sci.* **64**, 887–903 (2020).

48. R. Mozer, L. Miratrix, A. R. Kaufman, L. Jason Anastasopoulos, Matching with text data: An experimental evaluation of methods for matching documents and of measuring match quality. *Polit. Anal.* **28**, 445–468 (2020).

49. K. Keith, D. Jensen, B. O'Connor, Text and causal inference: A review of using text to remove confounding from causal estimates, in *Proceedings of the 58th Annual Meeting of the Association for Computational Linguistics*, (Online: Association for Computational Linguistics, 2020), pp. 5332–5344, https://doi.org/10.18653/v1/2020.acl-main.474.

50. J. Zhang, S. Mullainathan, C. Danescu-Niculescu-Mizil, Quantifying the causal effects of conversational tendencies, in *Proceedings of the ACM on Human-Computer Interaction 4* (CSCW2, 2020), pp. 1–24.

51. M. A. Cohen, R. T. Rust, S. Steen, "Measuring Perceptions of Appropriate Prison Sentences in the United States, 2000. ICPSR Version. Nashville, TN: Vanderbilt University [Producer], 2000." (Ann Arbor, MI: Inter-University Consortium for Political and Social Research.[distributor], 2004).

52. T. J. Leeper, MTurkR: Access to Amazon Mechanical Turk Requester API via r, 2017.

53. M. Costa, How responsive are political elites? A meta-analysis of experiments on public officials. *J. Exp. Political Sci.* **4**, 241–254 (2017).

54. D. M. Blei, Probabilistic topic models. *Commun. ACM* **55**, 77–84 (2012).

55. J. Hainmueller, D. J. Hopkins, T. Yamamoto, Causal inference in conjoint analysis: Understanding multidimensional choices via stated preference experiments. *Polit. Anal.* **22**, 1–30 (2013).

56. T. L. Griffiths, Z. Ghahramani, The Indian buffet process: An introduction and review. *J. Mach. Learn. Res.* **12**, 1185–1224 (2011).

57. F. Doshi, K. Miller, J. V. Gael, Y. W. Teh, Variational inference for the Indian buffet process, in *International Conference on Artificial Intelligence and Statistics* (AISTATS, 2009), pp. 137–144.
